# Regulatory Modules of Metabolites and Protein Phosphorylation in Arabidopsis Genotypes With Altered Sucrose Allocation

**DOI:** 10.3389/fpls.2022.891405

**Published:** 2022-05-19

**Authors:** Thorsten Stefan, Xu Na Wu, Youjun Zhang, Alisdair Fernie, Waltraud X. Schulze

**Affiliations:** ^1^Department of Plant Systems Biology, University of Hohenheim, Stuttgart, Germany; ^2^College for Life Science, Yunnan University, Kunming, China; ^3^Department of Central Metabolism, Max-Planck-Institute of Molecular Plant Physiology, Potsdam, Germany; ^4^Center of Plant System Biology and Biotechnology, Plovdiv, Bulgaria

**Keywords:** metabolites, phosphopeptides, co-expression analysis, clustering, network construction

## Abstract

Multi-omics data sets are increasingly being used for the interpretation of cellular processes in response to environmental cues. Especially, the posttranslational modification of proteins by phosphorylation is an important regulatory process affecting protein activity and/or localization, which, in turn, can have effects on metabolic processes and metabolite levels. Despite this importance, relationships between protein phosphorylation status and metabolite abundance remain largely underexplored. Here, we used a phosphoproteomics–metabolomics data set collected at the end of day and night in shoots and roots of Arabidopsis to propose regulatory relationships between protein phosphorylation and accumulation or allocation of metabolites. For this purpose, we introduced a novel, robust co-expression measure suited to the structure of our data sets, and we used this measure to construct metabolite-phosphopeptide networks. These networks were compared between wild type and plants with perturbations in key processes of sugar metabolism, namely, sucrose export (*sweet11/12* mutant) and starch synthesis (*pgm* mutant). The phosphopeptide–metabolite network turned out to be highly sensitive to perturbations in sugar metabolism. Specifically, KING1, the regulatory subunit of SnRK1, was identified as a primary candidate connecting protein phosphorylation status with metabolism. We additionally identified strong changes in the fatty acid network of the *sweet11/12* mutant, potentially resulting from a combination of fatty acid signaling and metabolic overflow reactions in response to high internal sucrose concentrations. Our results further suggest novel protein-metabolite relationships as candidates for future targeted research.

## Introduction

The ultimate goal of systems biology is to incorporate all interactions of molecular cellular components into a descriptive model that can also be predictive (Li and Snyder, 2011). To fully understand genotype-to-phenotype relationships at the systems level, comprehensive knowledge of the complex and dynamic interactions between transcripts, proteins, and metabolites that exist in an organism is required (Bassel et al., 2012). While recent improvements in “omics” technologies have facilitated a deeper understanding of the set of molecular interactions in plant cells, larger gaps in knowledge remain regarding interactions between proteins and metabolites. This is also partly because proteome analyses still suffer from lower coverage compared to transcript analyses. Thus, many studies focused on concordance between transcripts and changes in metabolite levels. While this often delivered valuable results (Brotman et al., 2012; Balmer et al., 2013; Rudd et al., 2015; Zhu et al., 2018), it must be used with care when inferring protein expression from transcriptome data, as transcript and protein abundance do not always correlate (Bassel et al., 2012; He et al., 2021). Also, establishing causation from transcript to metabolite concentrations is not always straightforward, as in many cases, correlations between metabolites and transcripts were found to be due to regulation of gene expression by metabolites rather than metabolite levels being changed as a consequence of change in gene expression (Gibon et al., 2006). Furthermore, changes in enzyme activities were often found to be strongly delayed compared to changes in transcript levels (Gibon et al., 2006).

However, despite the challenges in the analysis of the protein-metabolite interactome (PMI), such as the large diversity of small molecules or the lack of generally applicable approaches for system-wide monitoring of protein-metabolite interactions (Veyel et al., 2017), the benefits of a systematic investigation of interactions between proteins and metabolites may help to bridge the gap between genome-wide association studies and small-molecule screening studies (Li and Snyder, 2011). Small molecules not only represent cellular building blocks and metabolic intermediates but also function as regulatory ligands and signaling molecules that interact with proteins. In this way, alterations in concentrations of metabolites can affect cellular metabolism, growth, and development (Veyel et al., 2018). There is growing evidence that biological systems contain a multitude of small molecules/metabolites, many of which are still unexplored, which can form stable complexes with proteins. These are primary candidates with function in signaling and regulation (Li and Snyder, 2011), and together with cellular proteins, they form a still unexplored wealth of protein–metabolite interactions (Veyel et al., 2018).

It has, therefore, been suggested that proteomics and metabolomics are the methods of choice to study the qualitative and quantitative compositions of the constitutive cellular system (Arrivault et al., 2009; Feussner and Polle, 2016), as the proteome and the metabolome of plants do not just mirror transcriptional changes in response to environmental changes but also incorporate further processes, such as activation and deactivation of existing proteins by phosphorylations and dephosphorylations or reversible binding of other side groups. This might, in turn, trigger further signaling cascades (Feussner and Polle, 2016). Nevertheless, the analysis of the PMI received comparatively little attention, despite its high-potential importance for both basic research (e.g., identifying novel signaling molecules) and translational research (e.g., food security, bioenergy, and identification of lead compounds for drugs) (Veyel et al., 2017). An exception is the application of integrated omics analysis combining transcriptomics, proteomics, and metabolomics. Examples of such analysis are comparison of the interactomes of free-tillering and low-tillering wheat isolines (Wang et al., 2019) and construction of networks based on expression patterns of mRNAs on one hand and on co-expression of proteins including phosphorylation levels on the other, the integration of which substantially improved predictive power when inferring transcription factor activity (Walley et al., 2016).

Sucrose metabolism in plants is a unique and central metabolic pathway connected to long-term storage metabolism (starch), metabolic conversion (glycolysis), and, ultimately, generation of ATP during respiration. Sucrose is the primary transport form of carbohydrates in the phloem in the majority of plant species, and the loading of sucrose to the phloem is tightly regulated (Sauer and Stolz, 1994; Ward et al., 1997; Kühn et al., 1999; Liesche et al., 2011; Liesche, 2017). Furthermore, sucrose is a key metabolite in stress responses that accumulate in vacuoles in response, for example, to cold stress (Schulze et al., 2012). Thus, sucrose metabolism is an interesting pathway to explore protein-metabolite interactions and the dynamics of this network in a day-night cycle and upon additional perturbations.

Internal perturbations of sucrose concentrations can be achieved using mutants in metabolism or sucrose transport. The starch-less *pgm* mutant (Caspar et al., 1985) is known to have a high diurnal sucrose concentration in leaves but runs into carbon limitation during the night (Schulze et al., 1991, 1994). The *sweet11/12* mutant (Chen et al., 2011) is a sucrose export mutant. In contrast to the *pgm* mutant, *sweet11/12* has a high starch content in leaves that are not degraded at night (Chen et al., 2010). In the *sweet11/12* mutant, sucrose accumulation occurs in source tissues, while sink tissues experience sucrose starvation. These mutants provide valuable tools to study perturbations in the protein-metabolite regulatory network.

This study was performed based on a combination of phosphoproteome and metabolome profiling on shoots (source tissue) and roots (sink tissue). Making use of both mutants affecting carbon partitioning between metabolic pathways and allocation between plant tissues, we aimed to understand the relationships between protein phosphorylation and metabolite accumulation and allocation. Since phosphorylation is an important regulatory protein modification and proteins are the actual active components in cellular metabolism in the form of enzymes, transporters, or regulators, we expect to capture more direct relationships between protein activity status and metabolism. Results are expected to give new insights into the regulation of plant carbon status in a whole-plant context. Our final aim was the construction of metabolite-phosphopeptide networks for different genotypes (wild type and both mutants) to gain a deeper understanding of biochemical processes in relation to the cascade of changes that were triggered by modifying sucrose metabolism in the mutants.

## Methods

### Plant Materials and Growth Conditions

*Arabidopsis* seeds of wild-type (col-0), *pgm* mutant (Caspar et al., 1985) (point mutation mutant), and *sweet11/12* double mutant (crossing of the *sweet11* (*SALK_073269*) and *sweet12* (*SALK_031696)* T-DNA insertion lines) were imbibed and vernalized for 2 days and then germinated and grown under 12/12 day/night (22°C, 120 μE/s^*^m^2^) in $\frac{1}{2}$ MS medium in a hydroponic cultivation system (Schlesier et al., 2003). After 20 days, seedlings were starved by changing the growth medium to a sucrose-free medium and leaving the culture vessels under 8/16 day/night for 7 days. The light period was changed to a short day to induce carbon starvation metabolic phenotype in the *pgm* mutant. Shoots (leaves) and roots were harvested for microsomal protein preparation. Plants were harvested before the onset of flowering.

### Genotyping Analysis of T-DNA Mutants

Homozygous mutants were confirmed by PCR- and CAPS- (cleaved amplified polymorphic sequence) (Schaller and Oecking, 1999) based genotyping. The PCR amplification used T-DNA border primer LBb1.3 (5′-ATTTTGCCGATTTCGGAAC-3′) or LB4 (TGATCCATGTAGATTTCCCGGACATGA AG) and gene-specific primers (SIRK1-RP: 5′-TTTCCAGCATTTCCAACACTC-3′, SIRK1-LP: 5′-CACTAAGCTTGTTGAGGTCGC-3′; SAK1-RP: 5′-CAAACCAGGTCCATCAAGATC-3′, SAK1-LP: 5′-GAGATTCCGTCGCTTCTCTTC-3′; SAK2_wisc-RP TTCCATTCACTGCAGTCTGC, SAK2_wisc-LP GCAGAAGCTTTCAGCAATCC; SWEET11-RP TGAAGTGGGTGCTTTTGTTTC, SWEET11-LP CCGA AGAGTAATGTGACCACG; SWEET12-RP TCAAAGGCCAAAGCAATATACC, and SWEET12-LP ATGC AGGCCAACGTTCTATAG). The CAPS assays used gene-specific primers (PGM-LP AGGCTTCCGAGCA ACTCAATATC and PGM-RP CTGACCACTGCTGTAATTGAAC) to amplify DNA fragments that were digested with a restriction endonuclease BspCN I.

### Analysis of Primary Metabolites

Metabolite profiling of *Arabidopsis* seedlings was carried out by gas chromatography–mass spectrometry (ChromaTOF software, Pegasus driver 1.61; LECO) as described previously (Lisec et al., 2006) while using smaller amounts of reactants because of more sensitive equipment. Briefly, around 50 mg of plant materials were snap-frozen (< -60°C) and homogenized in a ball mill. After adding 1 ml of 100% methanol and 60 μl of ribitol, samples were mixed and centrifugalized. Supernatants were transferred to a glass vial, and 400 μl of chloroform and 600 μl of dH_2_O were added to the samples, which were then again centrifugalized. Then, 150 μl of supernatant was dried in a vacuum container, after which it was used for GC-MS measurement (a detailed description of extract preparation with larger amounts of reactants can be found in Figure 1 of Lisec et al., 2006). Samples were derivatized using a standard protocol of mass spectrometry-based untargeted plant metabolomics (Perez de Souza et al., 2019). Chromatograms and mass spectra were evaluated using TagFinder software (Luedemann et al., 2012). Metabolite identification was manually checked by mass spectral and retention index collection of the Golm Metabolome Database (Kopka et al., 2005). Peak heights of the mass fragments were normalized on the basis of the fresh weight of the sample and the added amount of an internal standard (ribitol). Statistical differences between groups were analyzed by Student's *t*-tests on the normalized data. Results were determined to be statistically different at a probability level of *P* < 0.05. Identified metabolites and their log_2_-transformed concentrations relative to the standard substance are available (Supplementary Table 1).

### Protein Preparation, Tryptic Digestion, and Phosphopeptide Enrichment

Microsomal membrane preparation and phosphopeptide enrichment were performed as described in the “ShortPhos” workflow (Wu et al., 2017). A total of 1–1.5 g of roots and shoots (fresh weight) was homogenized in a 10-ml extraction buffer (330 mM mannitol, 100 mM KCl, 1 mM EDTA, 50 mM Tris-MES, fresh 5 mM DTT, and 1 mM phenylmethylsulfonylfluoride, pH 7.5) in the presence of a 0.5% v/v proteinase inhibitor mixture (Sigma-Aldrich, Germany) and phosphatase inhibitors (25 mM NaF, 1 mM Na_3_VO_4_, 1 mM benzamidin, and 3 μM leupeptin) in Dounce Homogenizers. The homogenate was centrifuged for 15 min at 7,500 × g at 4°C. The pellet was discarded, and the supernatant was centrifuged for 75 min at 48,000 × g at 4°C. The microsomal pellet was resuspended in 100 μl UTU (6 M urea and 2 M thiourea, pH 8). The soluble fraction in the supernatant was precipitated with three times volume ethanol plus 40 μl ml^−1^ of 2.5 M sodium acetate (pH 5) overnight, and then proteins were resuspended in 500 μl UTU. Protein concentrations were determined using a Bradford (Sigma–Aldrich, Germany) assay with BSA (bovine serum albumin) as the protein standard.

An amount of 150 μg protein was aliquoted separately for tryptic digestion and phosphopeptides enrichment. Microsomal membranes and soluble protein were subjected to disulfide bond reduction by DTT and alkylation by iodoacetamide before protein was predigested for 3 h with endoproteinase Lys-C (0.5 μg μl^−1^; Wako Chemicals, Neuss, Germany) at room temperature. After 4-fold dilution with 10 mM Tris-HCl (pH 8), samples were digested with 3 μl sequencing-grade modified trypsin (0.5 μg μl^−1^; Promega) overnight at 37°C. After overnight digestion, 10% v/v trifluoroacetic acid (TFA) was added (until the pH was 3 or less) to stop digestion. Digested peptides were dried in a vacuum concentrator.

Dry peptides were dissolved in 200 μl of 1 M glycolic acid in 80% v/v acetonitrile (ACN) and 5% v/v trifluoroacetic acid (TFA). Phosphopeptides were enriched over titanium dioxide (TiO_2_) (GL Sciences, Japan). TiO_2_ beads (1.5 mg per sample) were washed once with 100 μl of 1% v/v ammonia solution and equilibrated three times with 50 μl of 1 M glycolic acid in 80% v/v ACN and 6% v/v TFA. The amount of 200 μl digested peptides was mixed with equilibrated TiO_2_ for 30 min incubation. Peptides and TiO_2_ bead mixture were washed one time with 100 μl of 1 M glycolic acid in 80% v/v ACN and 6% v/v TFA, and three times with 100 μl of 80% v/v ACN and 1% v/v TFA. Phosphopeptides were eluted from TiO_2_ beads three times with 1% v/v ammonia solution. Eluates were immediately acidified with 70 μl of 10% v/v formic acid. Acidified phosphopeptides were desalted over a C18 stage tip prior to mass spectrometric analysis (Rappsilber et al., 2003).

### LC-MS/MS of Peptides and Phosphopeptides

Enriched phosphopeptides were resuspended in 5 μl resuspension buffer (0.2% v/v TFA, 5% v/v ACN) and analyzed by LC-MS/MS using the standard setting as described (Wu et al., 2017) with nanoflow EASY-nLC™ 1200 System (Thermo Scientific, Germany) as an HPLC system and an Orbitrap hybrid mass spectrometer (Q Exactive™, Hybrid Quadrupole-Orbitrap™; Thermo Scientific, Germany) as a mass analyzer. Peptides were eluted from a 75 μm × 25 cm analytical column (EasySpray ES802; Thermo Scientific, Germany) on a linear gradient running from 5 to 90% acetonitrile over 180 min and sprayed directly into the Q-Exactive mass spectrometer. Peptides were identified *via* the MS/MS based on the information-dependent acquisition of fragmentation spectra of multiple charged peptides. Up to 12 data-dependent MS/MS spectra were acquired for each full-scan spectrum acquired at 70,000 full-width at m/z 200 resolution.

### Protein Identification and Ion Intensity Quantitation

Raw data acquired with the mass spectrometer were processed using MaxQuant version 1.5.3.8 (Cox and Mann, 2008). Spectra were matched against the Arabidopsis proteome (TAIR10, 35,386 entries) using the Andromeda search engine (Cox et al., 2011). Common contaminants (trypsin, keratin, etc.) were included during database searches. Carbamidomethylation of cysteine was set as a fixed modification, and oxidized methionine (M), acetylation (protein N-term), and phosphorylation (STY) were set as variable modifications. Trypsin was specified as the digesting protease, and up to two missed cleavages were allowed. The mass tolerance for the database search was set based on the default settings in MaxQuant with 4.5 ppm for full scans and 20 ppm for fragment ions. The multiplicity was set to 1. For label-free quantitation (LFQ), retention time matching between runs was chosen within a time window of 1 min. False discovery rate cutoffs were set to 0.01 for peptide and protein identification and to 0.01 for phosphorylation site assignment. The location of phosphorylation sites was determined with the site-scanning algorithm in Andromeda. Hits to contaminants (e.g., keratins) and reverse hits identified by MaxQuant were excluded from further analysis. Phosphopeptides (Supplementary Table 2), including their spectra, were submitted to the phosphorylation site database PhosPhAt 4.0 and are publicly available. The mass spectrometry proteomics data have been deposited to the ProteomeXchange Consortium *via* the PRIDE partner repository (Deutsch et al., 2017) with the dataset identifier PXD031942. Label-free quantitation was performed using LFQ-values calculated by MaxQuant (Cox et al., 2014), and further analysis was performed with the Perseus software (version 1.5.6.0) (Tyanova et al., 2016). Reported label-free intensity values (Phospho(STY)Sites.txt) were used for data analysis. Missing values were imputed from a normal distribution around the detection limit of the mass spectrometer as offered by the default settings in Perseus. For each peptide, imputed values from three biological replicates were averaged. Annotations were extracted from mapman (Thimm et al., 2004), and subcellular locations were obtained from SUBA3 (Tanz et al., 2013).

### Statistical Analysis

Metabolite concentrations and phosphopeptide intensities were measured for each of four conditions, “end of day/root” (DR), “end of day/shoot” (DS), “end of night/root” (NR), and “end of night/shoot” (NS), and in each condition, between four and six biological replicates were analyzed. All measurements were log_2_-transformed, and transformed values were used for further analysis. For comparisons of changes in metabolite and phosphopeptide levels between genotypes, log_2_-fold changes were calculated based on the original values.

Untransformed and transformed measurements were subjected to outlier classification using a Python function that classifies points as outliers by means of a modified z-score based on the median absolute deviation (MAD) (Iglewicz and Hoaglin, 1993). A threshold of 5 for the modified z-score was used, and any measurement that exceeded this threshold was removed. The threshold value was deliberately chosen conservatively, as due to the rather low number of measurements for each condition, we only removed rather obvious outliers. Descriptive statistical operations were performed using the “stats” module of SciPy. In particular, the procedures “describe,” “tmean,” and “tstd” were used to calculate means, variances, and standard deviations, whereas “median_abs_deviation” was used to compute the median over the absolute deviations from the median (MAD), and “kruskal” was used to calculate the *p*-value of a Kruskal–Wallis test by ranks. Other values, such as the ratio of the variance between conditions to the variance within conditions, *V*_*btw*_*/V*_*within*_, were calculated directly from the respective values.

### Clustering

Clustering was performed on the standardized mean metabolite concentrations (z-scores) of the four conditions, DR, DS, NR, and NS, separately for each genotype using the k-means clustering algorithm of scikit-learn (k-means++ initialization method, 100 runs with different centroid seeds, max. 300 iterations for a single run) and orange 3.25.1, as well as the hierarchical and k-means clustering algorithms of orange 3.25.1. In addition, clusters were calculated for genotype-overlapping concentration patterns, again using both hierarchical and k-means clustering. The arithmetic mean of each condition was calculated from the z-scores of all mean metabolite concentrations, therefore obtaining “average” patterns for the genotype-specific and genotype-overlapping clusters, respectively. The clusters obtained with this procedure were used to determine whether the standardized concentration pattern of a metabolite showed a qualitatively different behavior between genotypes. For hierarchical clustering, the Euclidean distance metric was used. The same clustering procedures were also applied to the standardized mean phosphopeptide intensities.

### Measuring Concordant Behavior of a Metabolite–Phosphopeptide Pair

To classify whether a metabolite-phosphopeptide pair qualitatively displays the same pattern, we developed a novel co-expression measure subsequently called “concordance index (*I*_*C*_)”. This index is based on the calculation of the pairwise deviations between measurement means for conditions DR, DS, NR, and NS relative to the standard deviation of the first member of the pair. For example, the pairwise deviation between conditions DR and DS, relative to the standard deviation of DR, was calculated by dividing the difference between the mean values of the measurements for DS and DR by the sample standard deviation of the measurements for DR (Equation 1). Pairwise deviations were calculated for all metabolites and phosphopeptides, resulting in 12 values overall for each metabolite or phosphopeptide.


(1) $$\begin{array}{r} d_{k}^{\left(M\right)}=\frac{{\bar{x}}_{j}-{\bar{x}}_{i}}{{sd\left(x\right)}_{i}} \end{array}$$

where, *i* and *j*(1 ≤ *i* ≤ 4, 1 ≤ *j* ≤ 4, *i*≠*j*) represent the conditions DR, DS, NR, and NS; *x*_i_ and *x*_j_ are the mean values of the measurements for conditions *i* and *j*, and *sd*(*x*)_*i*_ is standard deviation of the measurements of the condition *i*. The deviation $d_{k}^{\left(M\right)}$ is the *k*^th^ deviation, and 1 ≤ *k* ≤ 12 for the metabolites. Deviations between conditions $d_{k}^{\left(P\right)}$for the phosphopeptides were calculated accordingly.

To obtain the concordance indices, we first calculated the combined condition deviations $z_{k}^{\left(M,\;P\right)}$(Equation 2):


(2) $$\begin{array}{l} z_{k}^{\left(M,\;P\right)}=\\ sign\left(d_{k}^{\left(M\right)}\right)\;\cdot \;sign\left(d_{k}^{\left(P\right)}\right).\min\left(abs\left(d_{k}^{\left(M\right)}\right),\;abs\left(d_{k}^{\left(P\right)}\right)\right) \end{array}$$

The combined condition deviations $z_{k}^{\left(M,\;P\right)}$ were subsequently discretized into “deviation classes” (Table 1), which are mapped to contributions characterizing the strength of the concordant behavior between any two conditions. By determining the class that comprises $z_{k}^{\left(M,\;P\right)}$ for each *k*, we, thus, obtained the discretized combined condition deviations, or concordance index components, ${\tilde{z}}_{k}^{\left(M,\;P\right)}$. These concordance index components ${\tilde{z}}_{k}^{\left(M,\;P\right)}$ were added up, separately for negative and positive values of ${\tilde{z}}_{k}^{\left(M,\;P\right)}$, yielding the concordance indices $I_{C\left(-\right)}^{\left(M,\;P\right)}$(Equation 3) and $I_{C\left(+\right)}^{\left(M,\;P\right)}$ (Equation 4):


(3) $$I_{C\left(-\right)}^{\left(M,\;P\right)}=\sum_{k=1}^{12}{\tilde{z}}_{k}^{\left(M,\;P\right)}\cdot \;\delta ,\;\delta =\{\begin{array}{c} 1\;if\;{\tilde{z}}_{k}^{\left(M,\;P\right)}<0\\ 0\;if\;{\tilde{z}}_{k}^{\left(M,\;P\right)}\geq 0 \end{array}$$

(4) $$I_{C\left(+\right)}^{\left(M,\;P\right)}=\sum_{k=1}^{12}{\tilde{z}}_{k}^{\left(M,\;P\right)}\;\cdot \delta ,\;\delta =\{\begin{array}{c} 1\;if\;{\tilde{z}}_{k}^{\left(M,\;P\right)}>0\\ 0\;if\;{\tilde{z}}_{k}^{\left(M,\;P\right)}\leq 0 \end{array}$$

**Figure 1:**
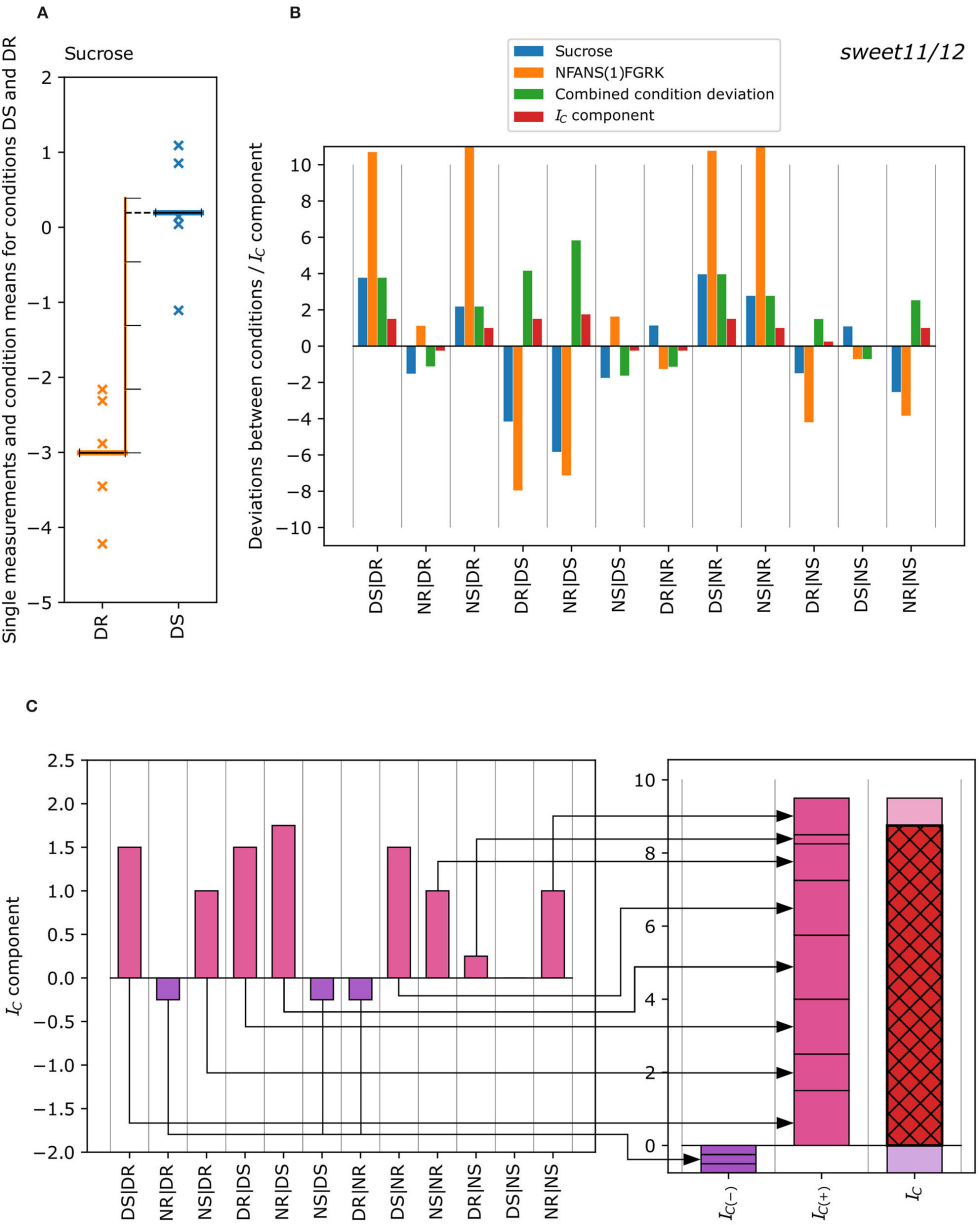
Exemplified calculation of the concordance index *I*_*C*_ of a metabolite-phosphopeptide pair from the single measurements of each condition. **(A)** Deviation of the mean of the single metabolite measurements for conditions DS and DR (DS|DR) in the *sweet11/12* mutant is measured in terms of the number of standard deviations (see Equation 1) with the mean of DS being ~3.8 standard deviations of DR higher than the mean of DR. **(B)** Difference in the condition means, where the blue bar of the first column (DS|DR) corresponds to a difference of +3.8 standard deviations for sucrose. The same calculation was performed for all differences between possible condition pairs for metabolites and phosphopeptides. These “condition deviations” are now compared for each metabolite-phosphopeptide pair (blue bars: metabolite; orange bars: phosphopeptide), resulting in the “combined condition deviations” (green bars; see Equation 2). It corresponds to lower absolute values of the condition deviations of metabolites and phosphopeptides and shows a positive sign in case deviations are either both positive or both negative, or a negative sign otherwise. The “combined condition deviations” are converted into discrete “deviation classes” (Table 1), resulting in concordance index components (red bars). **(C)** The concordance index components are summed up separately for negative (violet) and positive (pink) components (Equation 3) to yield the negative and positive concordance indices *I*_*C*(−)_ (violet) and *I*_*C*(+)_ (pink), which in turn were summed up to the concordance index *I*_*C*_ (red with black cross-hatch; Equation 4). The *I*_*C*_ is 8, and was calculated for the sucrose- NFANS(1)FGRK pair (*sweet11/12* mutant).

The negative and positive concordance indices were summed up to result in the final combined concordance index $I_{C}^{\left(M,\;P\right)}=I_{C\left(-\right)}^{\left(M,\;P\right)}+I_{C\left(+\right)}^{\left(M,\;P\right)}$. The derivation of the concordance index from the single measurements is visually explained in Figure 1.

**Table 1:** Classes, boundaries (with respect to $z_{k}^{\left(M,\;P\right)}$), and concordance index components ${\tilde{z}}_{k}^{\left(M,\;P\right)}$ for non-negative values of $z_{k}^{\left(M,\;P\right)}$.

**Class**	**Class 1**	**Class 2**	**Class 3**	**Class 4**	**Class 5**
Boundaries for $z_{k}^{\left(M,\;P\right)}$	[1, 2)	[2, 3)	[3, 5)	[5, 10)	[10, ∞)
Concordance index component ${\tilde{z}}_{k}^{\left(M,\;P\right)}$	0.25	1	1.5	1.75	2

*For negative values of $z_{k}^{\left(M,\;P\right)}$, corresponding negative boundaries were used, and the concordance index components ${\tilde{z}}_{k}^{\left(M,\;P\right)}$ were assigned the respective values of this table, but with a negative sign*.

To supplement the concordance index, we used Spearman's rank correlation coefficient to filter out the metabolite-phosphopeptide pairs with no strong support for a significant rank correlation between the respective measurements (α > 5%). In addition, we removed interactions where the Kruskal-Wallis test does not indicate significant differences between the conditions (resulting in a “flat” pattern) for either the metabolite or the phosphopeptide of the interaction pair. As a result, we arrived at a list with the most probable metabolite–phosphopeptides relationships for the wild type and the two mutants.

### Assessment of False-Positive Rate and Cutoff Values for Co-expression Analysis

To minimize false-positive co-expressions, we compared the share of metabolite-phosphopeptide pairs of both co-expression measures used (the concordance index *I*_*C*_ as well as Spearman's rank correlation coefficient ρ) between experimental and simulated data. We used three different modes to replace the experimental data by randomly drawn data sets (“simulation types”): (i) we randomly drew the metabolite values and calculated the co-expression measures of these to the experimental phosphopeptide measurements; (ii) we calculated the co-expression measures from the experimental measurements for the metabolites and randomly drawn values for the phosphopeptides; and (iii) we randomly drew metabolite and phosphopeptide values and calculated the co-expression from these two simulated data sets. In all cases where random values were drawn, we first drew values as condition means of a single molecule (metabolite or phosphopeptide) from a fitted Gamma distribution with location, shape, and scale parameter calculated from the experimental data of this molecule. Gamma distribution was preferred over normal distribution, as it is more flexible because of the three parameters used. Furthermore, the average distribution of phosphopeptide condition means is strongly asymmetrical, which often results in poor fit when using the normal distribution.

Using these drawn condition means, we then drew single random measurements for each molecule and each condition from a normal distribution based on the experimental condition mean and standard deviation of the respective molecule (assumption of normality of residuals). For each condition, the number of drawn measurements matched the experimental number of measurements. This procedure resulted in a close match of average standard deviations of condition means between experimental and simulated data sets for all genotypes, and both metabolites and phosphopeptides (deviation <1.1% in all cases), demonstrating good comparability.

### Over-representation Profiles

We calculated the overrepresentation of an entity within a sorted list (such as a metabolite within a list of metabolite-phosphopeptide interactions) by Fisher's exact test and applying Bonferroni's correction. The elements of the 2 × 2 contingency table used in Fisher's exact test were:


(5) $$\begin{array}{r} \left(\begin{array}{c} n\;-\;k\;k\\ \left(N\;-\;K\right)-\left(n-k\right)\;K\;-\;k \end{array}\right) \end{array}$$

Here, *n* is the current position on the sorted list, starting with the first position, *N* is the length of the list, while *k* and *K* are the numbers of occurrences of the entity of interest within the first *n* entries and in the entire list, respectively. We varied *n* from 1 to either the number of entries in the top 10% or the top 25% of the list and calculated the respective *p*-value using Equation (5) together with Bonferroni's correction, therefore arriving at a profile that can be represented visually. If an entity is overrepresented over a substantial section of the profile, then we can be more confident that we observe a real effect as opposed to only considering single values for *n*, where overrepresentation might just be observed by chance for a particular *n*. Using profiles can, therefore, be considered a more robust approach to quantifying overrepresentation.

Overrepresentation of Mapman functional categories (simplified to highest order categories and subsequently referred to as “bins”) (Thimm et al., 2004) in the concordance index-sorted metabolite-phosphopeptide interaction table was quantified by applying weights to the –log_10_ *p*-values. Specifically, we calculated the scalar product of the weights vector (*w*) with the vector of the –log_10_ *p*-values (*v*) for every point of the profile. This yields a value $x_{k}=w^{T}v$ for each bin, and the *x*_*k*_ can subsequently be ranked to obtain a list of relative over-representation for each bin in the interaction table. We chose the weights vector *w*^*T*^ = (*n, n*−1, …, 1), where *n* is the chosen number of interactions that represents the top of the interaction table. For positive interactions, we selected the *n* interactions with highest co-expression measures. As we considered the top 10% of all 42·3330 = 139, 860 metabolite-phosphopeptide interactions, we set *n* = 13, 986 in this case. Applying the chosen *w*^*T*^ resulted in bins that were over-represented among the first few highest interactions being assigned a higher weight than those that were over-represented towards the bottom of the table of the *n* top interactions.

Using the same approach, the overrepresentation of protein functional categories (“bins”) was calculated among the phosphopeptides and for genotype distances between phosphopeptides (in both cases, we considered the top 25% of all 3,330 ranked phosphopeptides, i.e., we set *n* = 832). Furthermore, the overrepresentation of metabolites and the overrepresentation of bins were calculated among the metabolite-phosphopeptide interaction pairs. The results were finally normalized to the range (0, 1) (“scaled ranks” ρ_*SC*_) and summarized in Supplementary Table 3. Ranking was performed for each genotype (wild type, *pgm* mutant, and *sweet11/12* mutant) as described above, and the scaled rank tables of bins and metabolites were finally sorted according to their mean scaled rank ρ_*SC*_ over all genotypes in ascending order. Bins and metabolites with lower rank values were more overrepresented. All initial tables (phosphopeptide abundance table, interaction table, and genotype distance table) were sorted in descending and ascending orders prior to overrepresentation analysis, resulting in ranked lists of overrepresented bins or metabolites.

## Results

**Figure 2:**
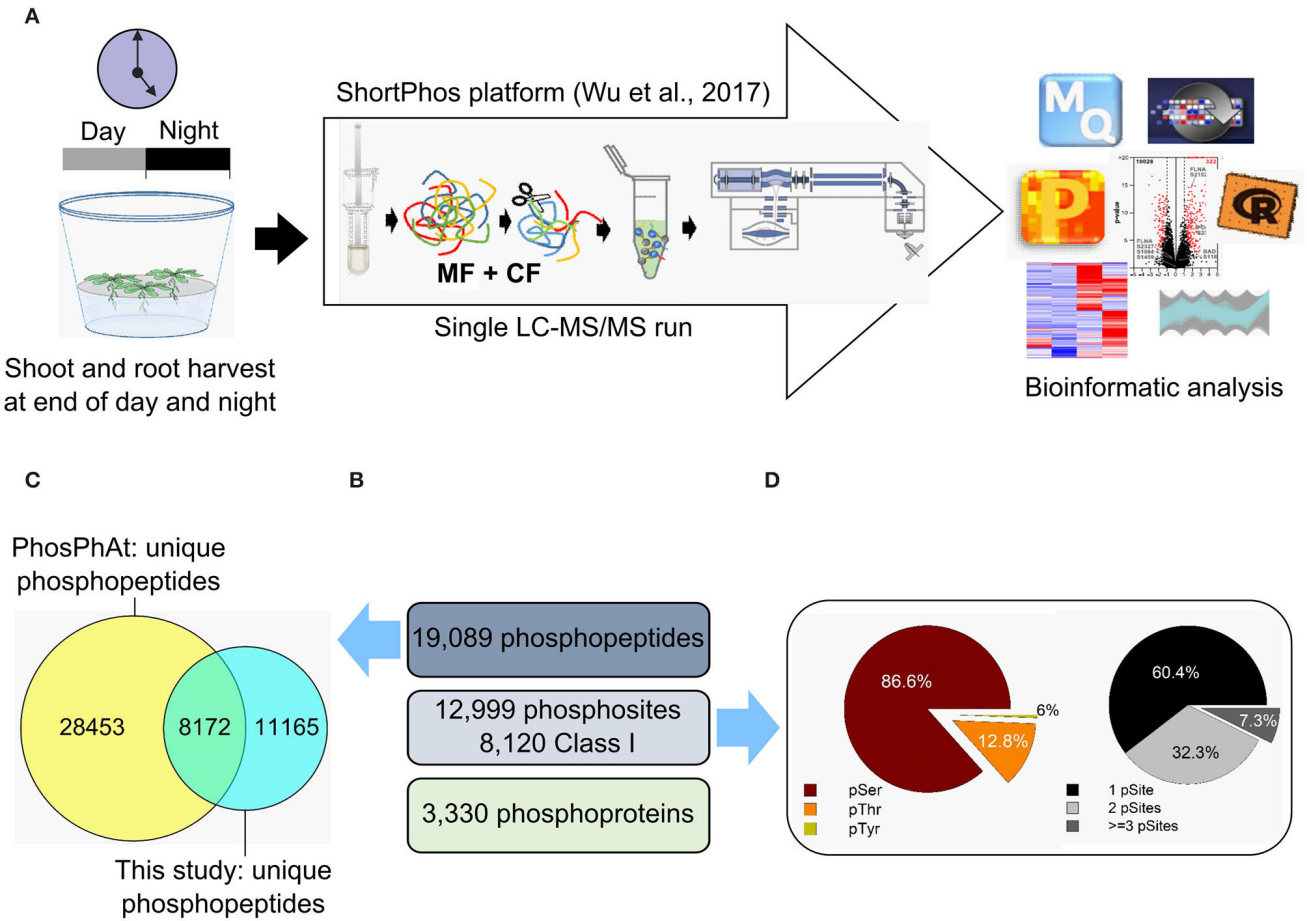
Phosphoproteome analysis of *Arabidopsis thaliana* shoots and roots. **(A)** Samples were collected at the end of the day and at the end of the night. Proteins were isolated, and phosphopeptides were enriched using the ShortPhos platform (Wu et al., 2017). The phosphopeptides were measured in a single-run mode using Q-Exactive and identified with MaxQuant following data analysis with Perseus. **(B)** The number of unique phosphopeptides, phosphosites, and phosphoproteins identified in our phosphoproteome of *Arabidopsis thaliana* shoots and roots. **(C)** The unique phosphopeptides identified in our phosphoproteome were compared with those deposited in the PhosPhAt database. **(D)** Distribution of the amino acid residues (left) and frequency of phosphorylated residues (right).

Phosphopeptides and metabolites were analyzed in the shoot and root tissue of each genotype at the end of the day (1 h before dark) and at the end of the night (1 h before light) after growth in short-day condition (8/16-h light/dark cycles). The same tissue was used for metabolic profiling as well as for phosphoproteomic analysis (Figure 2A) on four to six biological replicates. In total, 42 metabolites were quantified (Supplementary Table 1). These were 6 fatty acids, 17 proteinogenic and 2 non-proteinogenic amino acids, 6 other organic acids, 5 sugars, 3 sugar alcohols, one inorganic acid (phosphoric acid), one phosphoric ester (glycerol-3-phosphate), and one polyamine (putrescine).

A total of 19,089 unique phosphopeptides matching 5,019 proteins were identified by Maxquant using an accepted FDR of 0.01% (Figure 2B). This is a deep coverage of the phosphoproteome achieved by single-step purification (Wu et al., 2017). There was an overlap of 8,172 unique phosphosites between this study and the PhosPhAt 4.0 database (Figure 2C). Most of the identified phosphorylated amino acids were serines (83.8%), followed by threonines (15.5%) and tyrosines (0.7%) (Figure 2D). The phosphopeptides were distributed to 54.5% single, 34.9% double, and 10.6% triple phosphorylated peptides (Figure 2D). A total of 3,330 phosphopeptides were used for quantitative analysis (Supplementary Table 2) across all harvesting time points and tissues in each genotype. In order to perform statistical analyses, the phosphopeptides used for quantitative analysis were required to have LFQ values in at least three biological replicates and to be present in at least two of the sampled conditions (DS, DR, NS, and NR).

We analyzed all metabolite-phosphopeptide pairs for concordant behavior, i.e., similar concentration patterns in shoots and roots at the end of the day and at the end of the night. To assess the similarity of patterns, we looked for significant differences between any two conditions using a novel “concordance index”, (*I*_*C*_), described in the methods section. The concordance index *I*_*C*_ represents a smoothed similarity score to describe concordance or discordance between metabolite and phosphopeptide patterns. This index was complemented by Spearman's rank correlation coefficient ρ to extract pairs with a substantial probability of concordance, which were then assembled to form metabolite-phosphopeptide interaction networks for each genotype.

### Concordance Index as a Robust Measure to Describe Pattern Similarity

To measure the analogous (concordant) behavior of a metabolite-phosphopeptide pair, the “concordance index” *I*_*C*_ was calculated (see Equations 1–4; Figure 1). This index aimed to quantify how well the mean changes in the measured concentrations of the metabolite and the measured intensities of the phosphopeptide match over the four conditions, DR, DS, NR, and NS, with changes either in the same direction (analogous to a positive correlation) or in the opposite direction (analogous to a negative correlation). We expect that such a pattern-based index will give a more reliable estimate for the concordant behavior of a metabolite–phosphopeptide pair than a (rank-based) correlation measure, as it awards substantial differences between conditions instead of similar order or ranks of measurements. The latter might carry a considerable amount of randomness in case of small within-condition variation. Using the concordance index also overcame another issue with correlation measures: if measurements form two data clusters, with very little variation within each cluster, but a large difference between the clusters (i.e., if metabolites or phosphopeptides differ largely just between tissue or time of day), then a regression line will connect two narrow point clouds, and correlation measures might be very high despite only limited evidence of concordant behavior of the metabolite–phosphopeptide pair (Supplementary Figure 1).

**Figure 3:**
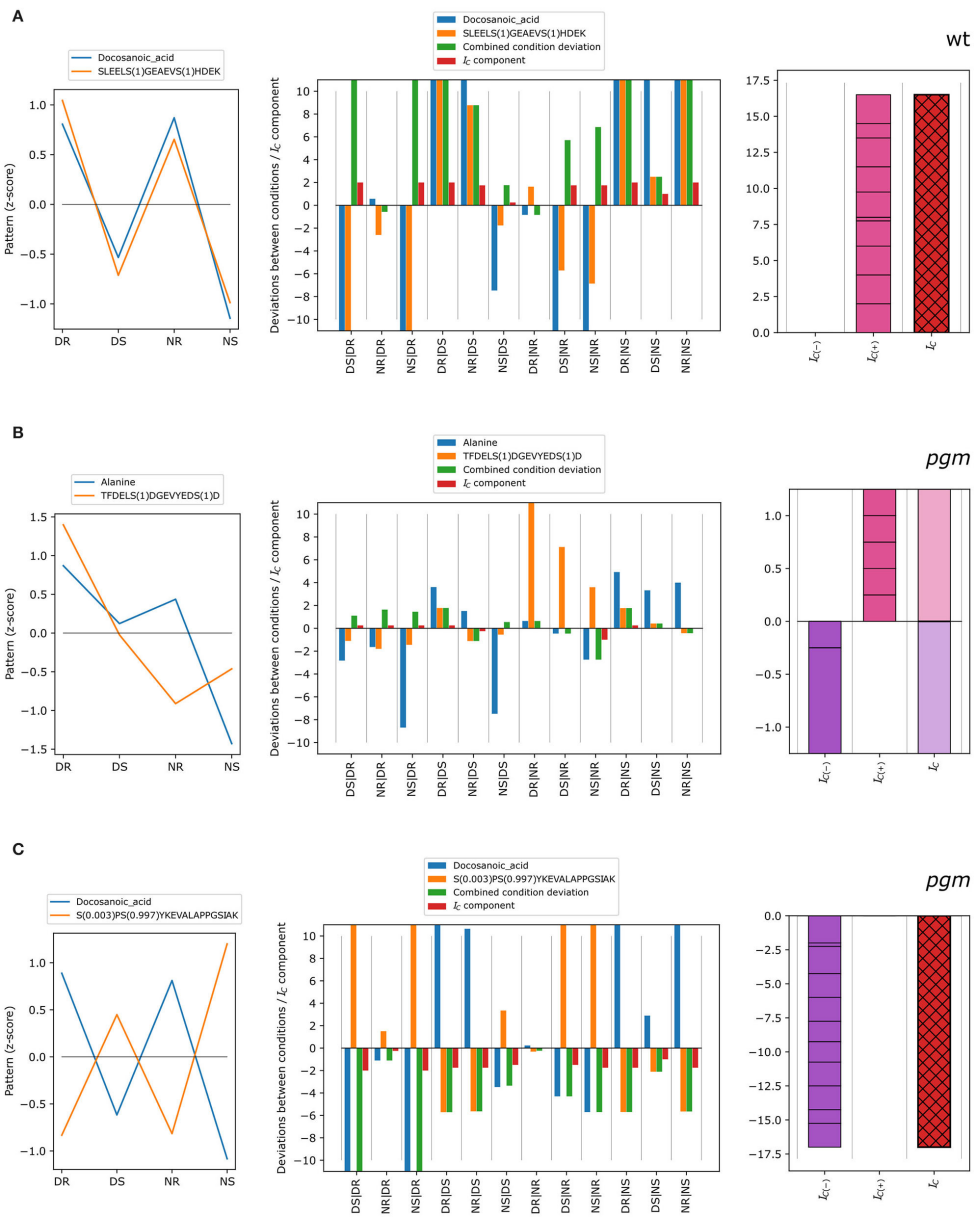
Different types of concordant behavior of metabolite-phosphopeptide pairs over the four conditions: DR, DS, NR, and NS. **(A)** Example of positive concordance between metabolites and phosphopeptides. **(B)** Example of no concordance between metabolites and phosphopeptides. **(C)** Example of strongly negative concordance (discordance). The left figure in each panel shows the z-score pattern over the four conditions, DR, DS, NR, and NS, whereas the middle and right figures show the derivation of the concordance index *I*_*C*_ from the deviations between conditions as in Figure 1.

The concordance index was exemplified for different scenarios (Figure 3). In cases of concordant patterns of metabolite docosanoic acid and phosphopeptide SLEELS(1)GEAEVS(1)HDEK in wild type (Figure 3A), standardized condition means (z-scores) show a similar pattern with higher average measurements in roots compared to shoots, and with higher values for shoots at the end of the day than at the end of the night for both the metabolite and the phosphopeptide. Measured as multiples of the standard deviation of one of the conditions (see Equation 1), this translates into strongly concordant deviations between most condition pairs (Figure 3A, center): blue (metabolite, $d_{k}^{\left(M\right)}$) and orange (phosphopeptide, $d_{k}^{\left(P\right)}$) bars, have high absolute values and the same direction, resulting in positive concordance index components. These components are added to yield the negative and positive concordance indices, which in turn sum up to the concordance index (red with black cross hatch) of 16.5. In contrast, the z-score patterns of alanine and TFDELS(1)DGEVYEDS(1)D in the *pgm* mutant (Figure 3B) showed little similarities, as some components of the concordance index are positive while the others are negative (Figure 3B, center), reflecting deviations between conditions in the same direction or in different directions, respectively. In total, the discretized deviations that sum up to negative (violet) and positive (pink) concordance indexes cancel each other out, resulting in no concordance (*I*_*C*_ = 0). In the case of docosanoic acid and phosphopeptide S (0.003)PS (0.997)YKEVALAPPGSIAK in the *pgm* mutant, the z-scores show opposite patterns (Figure 3C). Thus, most of the deviations between condition pairs (center; blue and orange bars) have an opposite sign, resulting in negative combined condition deviations and concordance index components (Figure 3C, center). The negative and positive concordance indices are therefore *I*_*C*(−)_ = −17 and *I*_*C*(+)_ = 0, giving a concordance index of −17, which is indicative of discordant co-expression patterns.

**Figure 4:**
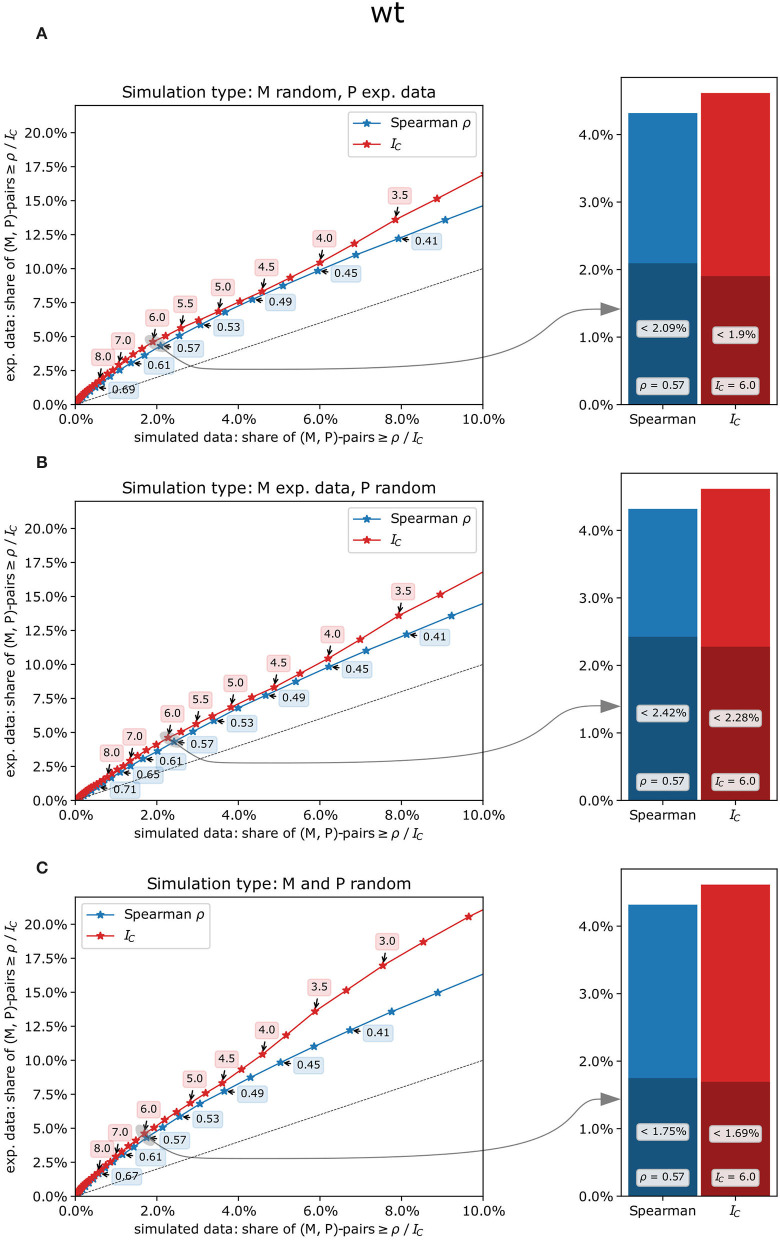
Comparison of experimental and simulated data sets contrasting the concordance index *I*_*C*_(red) with Spearman's rank correlation ρ (blue) in the wild type. **(A)** only the metabolites' measurements were randomly drawn, and co-expression measures were subsequently calculated using experimental measurements for the phosphopeptides, **(B)** only the phosphopeptides' measurements were randomly drawn, and **(C)** both metabolites' and phosphopeptides' measurements were randomly drawn. Left panels: percentage range of co-expression pairs exceeding respective threshold values in experimental data (y-axis) vs. simulated data (x-axis). Concordance indices *I*_*C*_ (red), which are discrete by definition, were examined for every possible value from −17.5 to 17.5 (i.e., −17.5, −17.25, −17.0, etc.), while Spearman's ρ (blue) was subdivided into 100 classes from −1 to +1. Dashed line: *y* = *x*, i.e., points above that line show a higher share of pairs in the experimental data set for the given threshold value than could be expected by chance. Right panels: share of metabolite-phosphopeptide pairs for *I*_*C*_ = 6 and Spearman's ρ =.57. The entire height of the bars represents the share of co-expression pairs in the experimental data set, and the dark-shaded bars represent the share of co-expression pairs in the simulated data set.

To estimate the likelihood of co-expression patterns to be genuine biological effects rather than random artifacts, the concordance index *I*_*C*_ and Spearman's ρ were calculated from experimental measurements of metabolites and phosphopeptides as well as from a randomized data set in which metabolites, phosphopeptides, or both were replaced by simulated data based on the respective experimental values (see methods, Supplementary Table 4). For all scenarios, both co-expression measures and all genotypes, higher shares of co-expression pairs at different threshold values were found in experimental data sets compared to simulated data (Figure 4). Thus, both co-expression measures resulted in a substantial proportion of likely true co-expressions of metabolites and phosphopeptides with an increasing likelihood for smaller shares of pairs from simulated data sets, especially for *I*_*C*_-values (Figure 4). For example, in the wild type, the share of co-expression pairs in experimental data with an *I*_*C*_-value of at least 6 is more than two times as high as than in simulated data for all three simulation types (Figure 4; Supplementary Table 4). Similar ratios were found for the other genotypes and for matching values of Spearman's ρ. For higher threshold values of *I*_*C*_ *or ρ*, these ratios even increase (Supplementary Table 4).

### Metabolomics Data Set

We compared the variance of each metabolite in the biological replicates analyzed (within-sample variance) to the variance between the four harvested samples (between-sample variance) for each genotype and condition (end of day root, DR; end of day shoot, DS; end of night root, NR; end of night shoot, NS). In the wild type, the fatty acids docosanoic acid, hexadecanoic acid, tetradecanoic acid and octadecanoic acid, the amino acids isoleucine and valine, and putrescine, nicotinic acid and fumaric acid showed the highest ratio of between-sample to within-sample variance, indicating that these metabolites showed great differences between tissues and/or day/night. In contrast, maltose, trehalose, and erythritol showed a relatively low variation between tissues and harvesting time points compared to the variation in samples in the wild type (Supplementary Table 1).

We grouped the metabolites into clusters to differentiate characteristic concentration patterns over the conditions DR, DS, NR, and NS. To avoid bias by considering “flat” patterns with a low ratio of between-sample to within-sample variance, only metabolites showing a significant difference (Kruskal-Wallis test with α = 0.05) between any two of the conditions DR, DS, NR, and NS were subjected to clustering. Metabolites displaying “flat” patterns were assigned to a single, separate cluster (cluster “Z”, Supplementary Table 5). To assess the robustness of the results, we performed both hierarchical and k-means clustering. In order to choose a suitable number of clusters, we used the silhouette score of k-means clustering and aimed for even cluster sizes and the identification of qualitatively sufficiently different patterns when using hierarchical clustering. In the case of the wild type, both the k-means and hierarchical clustering methods gave almost identical results when choosing 13 clusters for both hierarchical and k-means clustering (only nicotinic acid and tyrosine were assigned to different clusters). This configuration also gave relatively even cluster sizes (Supplementary Table 5, “MetClusters_WT”). The clustering was also stable with respect to the single genotypes, as for each genotype, similar clusters were obtained with the respective clustering method (Supplementary Table 5).

**Figure 5:**
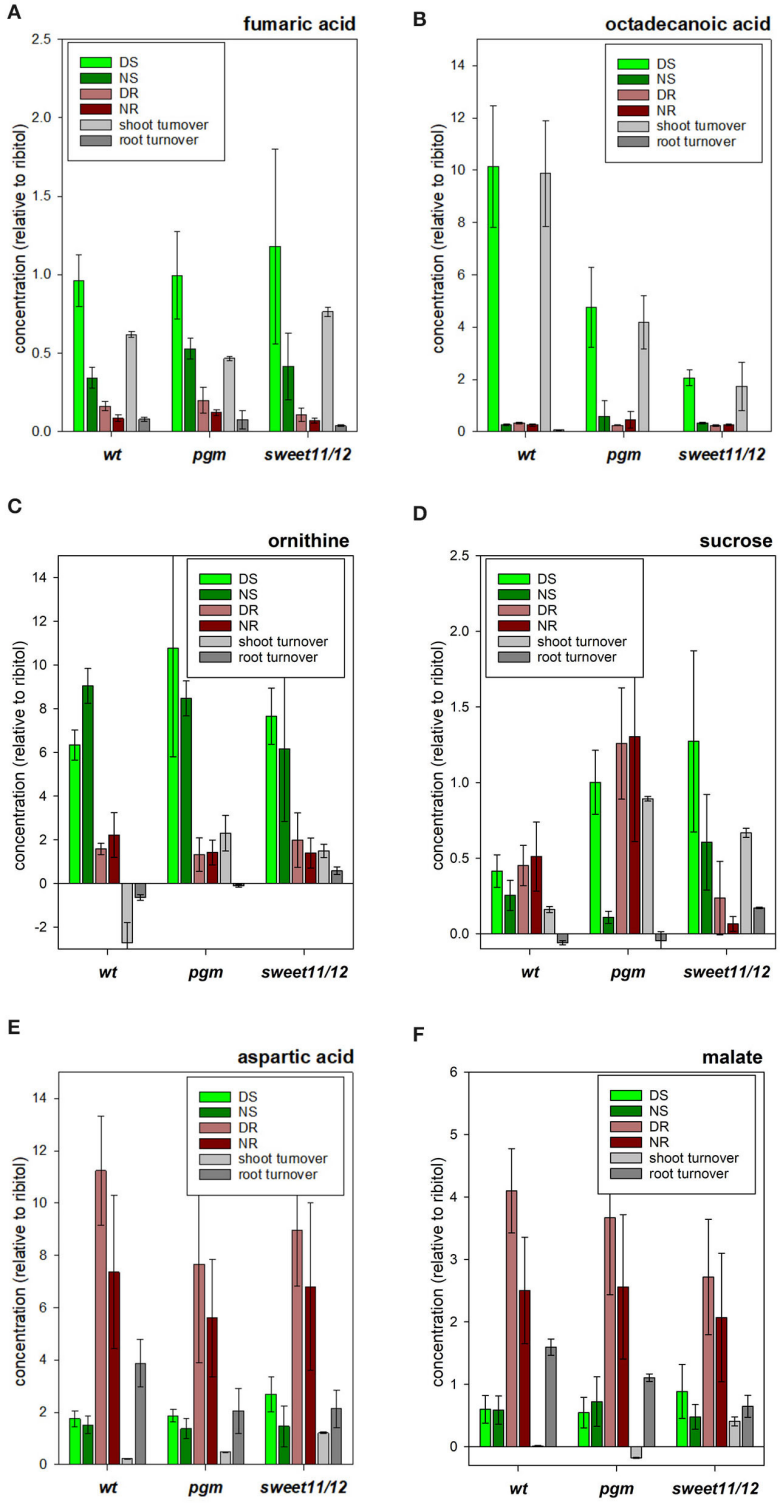
Concentrations of selected metabolites. **(A)** ornithine, **(B)** fumaric acid, **(C)** octadecanoic acid, **(D)** sucrose, **(E)** aspartic acid, and **(F)** malic acid in the different samples in the wild type, the *pgm* mutant, and the *sweet11/12* mutant as well as daily turnover rates. The turnover rates were calculated as the difference of concentrations at the end of the day and at the end of the night. Averages of five to six independent samples with standard deviations are shown.

Metabolites of clusters A_WT, D_WT, and F_WT in the hierarchical clustering approach (Supplementary Table 5, “MetClusters_WT”) containing fumaric acid (Figure 5A), asparagine, and octadecanoic acid (Figure 5B) were found with high concentrations in the shoots at the end of the day but with low concentrations in the roots at the end of the night. Putrescine, arginine, and ornithine (Figure 5C) were further examples of such metabolites with high concentrations in the shoot compared to the root with only small differences between day and night within each tissue. Metabolites in cluster B_WT showed high concentrations in the shoots compared to the roots with only small differences between day and night within each tissue. The third group of metabolites, corresponding to clusters G_WT and L_WT, comprised those with generally higher concentrations in the roots than in the shoots and higher levels at the end of the day than at the end of the night in roots. Sucrose (Figure 5D) and the amino acids valine, isoleucine, and aspartic acid (Figure 5E), and malic acid (Figure 5F) were in this group. Another group of metabolites, in cluster I_WT, showed strong differences between tissues and a high turnover in one of the tissues. For example, methionine and phosphoric acid were found with higher concentrations in the roots than in the shoots in general, while concentrations in the shoots at the end of the night were higher than those at the end of the day. *Myo*-inositol, being the only member of cluster J_WT in hierarchical clustering, showed high concentrations in the roots compared to in the shoots but with higher levels at the end of the night than at the end of the day in the roots. The sixth group of metabolites (cluster F_WT) showed higher concentrations during the day and lower concentrations at the end of the night but only small to medium differences between the roots and the shoots. Among these, asparagine, glycerol, and octanoic acid were prominent examples. Clustering results of all genotypes and details on the metabolites in the identified clusters are listed in Supplementary Table 5.

### Phosphoproteomics Data Set

When phosphopeptides were classified by their functional category according to Mapman (Thimm et al., 2004), two functional groups were overrepresented among the phosphopeptides with highest average abundance across all conditions (DR, DS, NR, and NS): Mapman bin 29.2 (protein.synthesis) was overrepresented among the first 50–100 most frequent phosphopeptides, while phosphopeptides classified within bin 31.4 (cell vesicle.transport) were overrepresented among the first 800 most frequent phosphopeptides (corresponding to the top 25% of the most abundant phosphopeptides, Supplementary Figure 2). These two bins were also the bins with lowest rank numbers (ρ_*SC*_, for calculation see methods) when calculating over-representation ranks, followed by bins 17.2 (hormone metabolism/auxin) and 30.5 (signaling/G-proteins) (Supplementary Table 3, ST3A), indicating high concentrations for many of the phosphoproteins in these groups. Scaled ranks of these bins were low not only in the wild type but also in the *pgm* and *sweet11/12* mutants (Supplementary Table 3, ST3A). Thus, ribosomal proteins (bin 29.2.2) were overrepresented among the most abundant proteins in the data set, reflecting the well-known high abundance of this protein group (Piques et al., 2009). In contrast, proteins with signaling functions (e.g., in bins 30.11 and 30.2), transporters (e.g., in bins 34.15, 34.2, and 34.21), and proteins involved in protein degradation (bin 29.5) were overrepresented in about 800 proteins with lowest average intensity, i.e., the lower-abundant proteins.

Based on the variance in the six biological replicates compared to the variance across the four samples, we identified phosphopeptides with especially high between-sample variance, i.e., those which showed strong differences between at least two sampling conditions. Protein functions among these phosphopeptides were, among others, signaling proteins, transporters, proteins of N-metabolism, and proteins of photosynthesis. Apparently, phosphorylation of these proteins showed marked differences between the roots and the shoot and/or day and night. In contrast, other signaling proteins, ribosomes, and proteins with developmental functions showed low between-sample variance (Supplementary Table 2).

### Mutants in Sucrose Partitioning and Allocation Have Altered Metabolite and Phosphorylation Patterns

The *pgm* mutant is deficient in starch synthesis. As a consequence, higher cytosolic sucrose levels in leaves at the end of the night were observed (Figure 5D). The high sugar levels previously were shown to result in higher respiration rates in the *pgm* mutant and reduced carbon allocation to roots and seeds (Schulze et al., 1991). As a consequence, the *pgm* mutant was characterized to run into carbon starvation during long and extended night periods, leading to slower root growth at night times (Yazdanbakhsh et al., 2011) and lower overall seed yield (Schulze et al., 1994). At the molecular cellular level, global alteration in gene transcription (Thimm et al., 2004) and polysome loading (Pal et al., 2013) were described. The *sweet11/12* mutant is deficient in sucrose export from the leaves (Chen et al., 2011). As a consequence, starch accumulates at high levels in source leaves, and less carbohydrates are available for growth in sink tissues such as roots and seeds. We analyzed the log_2_-fold changes in mutants vs. wild type for metabolite concentrations in each of the samples DR, DS, NR, and NS to assess whether the difference between genotypes is significant (Kruskal-Wallis test, see Supplementary Table 6, ST6A).

As expected, the sucrose in the *pgm* mutant significantly accumulated at higher concentrations than in the wild type during the day in the roots and shoots. At night, sucrose concentrations in the *pgm* mutant were significantly reduced in the shoots and roots (Figure 5D). In the *sweet11/12* mutant, higher than wild-type sucrose concentrations were found in the shoots at the end of the day as well as at the end of the night. In contrast, the root tissue in the *sweet11/12* mutant was highly depleted of sucrose, especially at night (Figure 5D). A similar pattern was observed for the sugars glucose, fructose, and maltose, with strong accumulation in shoot tissues at the end of the day and substantial depletion in root tissues at night.

The lack of sucrose export in the *sweet11/12* mutant coincided with the accumulation of maltose, *myo*-inositol, and fatty acids. These concentration changes were observed for octadecanoic acid, hexadecanoic acid, and tetradecanoic acid. In the wild type, these fatty acids were found to accumulate at high concentrations in the shoots during the day and were metabolized at night (Figure 5B). In the roots, long-chain fatty acids accumulated at night but at lower concentrations as in the leaves. In the *pgm* mutant (Figure 5B), and especially in the *sweet11/12* mutant (Figure 5B), the high turnover of long-chain fatty acids observed in wild type leaves was substantially reduced, with significantly lower than wild type concentrations during the day, and higher than wild type accumulation in shoots at night.

Several amino acids also showed different concentration patterns among the genotypes. For most of the amino acids, significantly higher concentrations in the shoots at the end of the day were found in the *sweet11/12* mutant than in the wild type. For some of the amino acids (serine, lysine, methionine, histidine, and ornithine), this was also true when comparing the *pgm* mutant to the wild type (Supplementary Table 6, ST6A).

Some of the organic acids showed qualitatively different patterns among the genotypes. A common feature for most of the organic acids was lower concentrations in the roots at the end of the day in the *sweet11/12* mutant compared to the wild type. For malic acid, fumaric acid (Figure 5A), succinic acid, and pyruvic acid, the differences were significant. Differences in organic acids between the *pgm* mutant and the wild type were less pronounced, with fumaric acid showing significantly increased concentrations at the end of the night in both tissues and with pyruvic acid showing significantly decreased concentrations in the shoots at the end of the day. Furthermore, the concentration of glycerol-3-phosphate was significantly higher in the *pgm* mutant in the shoots at the end of the night and was significantly lower in the shoots at the end of the day. Similarly, the polyamine putrescine shows a significant increase in the shoots at the end of the night in the *pgm* mutant, while in the *sweet11/12* mutant, it displayed a significantly lower concentration in the roots at the end of the day. Phosphoric acid concentration was significantly decreased in the *sweet11/12* mutant in both tissues at the end of the night (Supplementary Table 6, ST6A).

**Figure 6:**
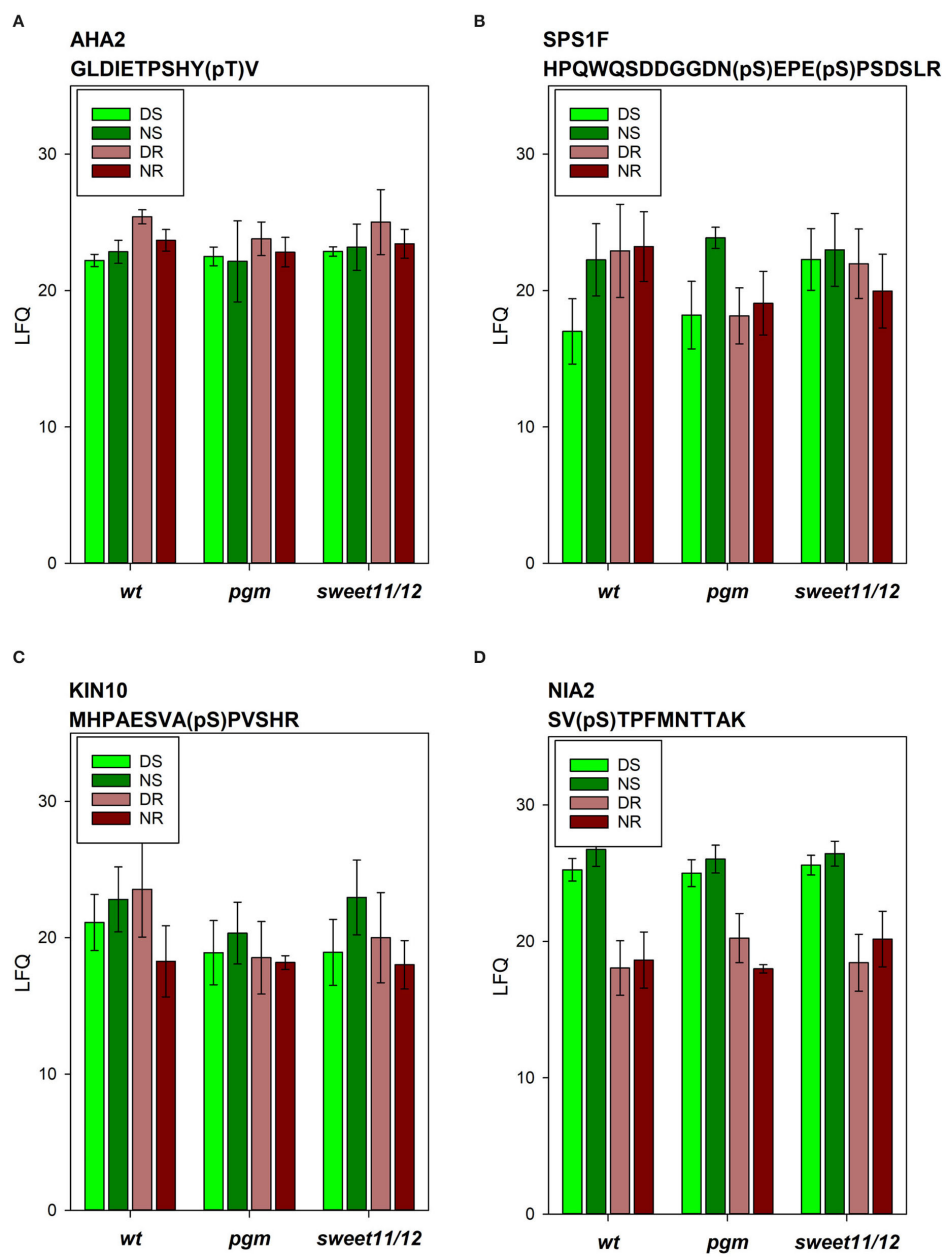
Phosphopeptide intensities of selected phosphopeptides. **(A)** Plasma membrane ATPase AHA2, **(B)** sucrose-phosphate synthase SPS1F, **(C)** kinase KIN10, and **(D)** nitrate reductase NIA2 in the different samples in the wild type, the *pgm* mutant, and the *sweet11/12* mutant. Averages of four to six independent samples with standard deviations are shown.

Examples of differences in protein phosphorylation levels across the genotypes were also found: among the phosphopeptides in the wild type, phosphorylation of AHA2 in activating T937 was highest in the roots at the end of the day and generally slightly higher in the roots than in the shoots (Figure 6A). In the *pgm* mutant, AHA2 phosphorylation in the roots at the end of the day was significantly reduced. In contrast, in both mutants, we observed increased phosphorylation of vacuolar ATPase subunit VHA-A3 in the shoots at the end of the day, which was significant in the *sweet11/12* mutant (Supplementary Table 6, ST6B). Generally, strong changes in phosphorylation patterns in mutants vs. wild types are observed especially in proteins involved in sucrose metabolism and signaling, such as sucrose-phosphate synthase (SPS1F; Figure 6B), cytosolic invertase (CINV1), kinase KIN10 (Figure 6C), and in a protein of nitrate assimilation (NIA2; Figure 6D).

### Genotype-Overlapping Clusters of Metabolites and Phosphopeptides

Assigning concentration patterns to genotype-overlapping clusters (i.e., the cumulative set of patterns of all the three genotypes, see Supplementary Tables 5, 7, “Clustering_OverAllGT”) gives an additional view with a focus on qualitative changes in patterns over the four conditions when comparing the three genotypes instead of quantifying the strength of changes between single conditions. Only patterns that were not “flat” (see methods section) were used for the analysis, while the “flat” patterns were aggregated in a separate cluster (named “Z” in Supplementary Tables 5, 7).

Using the genotype-overlapping clusters allowed us to assess which metabolites showed the largest qualitative changes between the genotypes regarding their patterns. We used the weighted number of changes between the genotypes for different clustering approaches (hierarchical and k-means with different numbers of clusters) and complemented this value with the weighted sum of the Euclidean distances between any two of the genotypes to arrive at a “between-genotype distance” *d*_*GT*_that provided us with a robust means for quantifying variation of patterns between the genotypes. Sorting the table of metabolites by this between-genotype distance *d*_*GT*_shows that the largest qualitative pattern changes between the genotypes were found for sucrose (Supplementary Table 5, Clustering_OverAllGT). A large difference between the genotypes was indeed expected for sucrose, as the mutants were explicitly chosen to cover different sucrose levels in the tissues and between night and day. Also, the sugar maltose and the amino acid tryptophan showed large divergences between the genotypes, with *d*_*GT*_ above 0.9, followed by glutamic acid, nonanoic acid, octanoic acid, and citric acids with *d*_*GT*_ above 0.8. The amino acids methionine, phenylalanine, histidine, serine, tyrosine, leucine, and proline as well as phosphoric acid and glycerol-3-phosphate all showed a *d*_*GT*_between 0.6 and 0.8, still indicating a qualitatively different behavior between the genotypes.

Clusters of the phosphopeptides were, in general, less clear-cut than the metabolite clusters, and the clusters were harder to demarcate. To cluster the phosphopeptides, we used the same clustering approach that was applied to the metabolites. Since the results indicated that the phosphopeptide clusters were harder to demarcate, we regarded possible conclusions from this analysis with more caution (Supplementary Table 7). In addition, we calculated the scaled ranks ρ_*SC*_ of the bins in terms of their overrepresentation in tables sorted by *d*_*GT*_in descending and ascending orders to determine bins with generally large pattern differences between genotypes, and those whose patterns show a rather similar behavior for all the genotypes (Supplementary Table 3, ST3G, ST3H). Proteins of carbohydrate metabolism (bins 2 and 3), calcium signaling proteins (bin 30.3), and proteins involved in protein targeting (bin 29.3) were among the protein categories with the highest between-genotype distance *d*_*GT*_. In particular, phosphopeptides IRS(1)EMQIWSEDDKSSR (AT4G10120, sucrose phosphate synthase, bin 2.1, *d*_*GT*_0.96), S(1)MS(1)ELS(0.964)T(0.034)GYS(0.001)R (AT1G35580, cytosolic invertase, bin 2.2, *d*_*GT*_ 0.93), S(1)YTNLLDLASGNFPVMGR (AT1G06410, trehalose phosphate synthase, bin 3.2, *d*_*GT*_0.88), and ASS(1)SVSTLYK (AT4G03550, callose synthase, bin 3.6, *d*_*GT*_0.81) were among the phosphopeptides with highest distances between the genotypes. Thus, it became apparent that the perturbation of sucrose metabolism in *pgm* and *sweet11/12* resulted in global changes in phosphorylation patterns, with proteins involved in pathways connected to sucrose metabolism being highly overrepresented.

### Networks Reconstructed From Concordant Behavior of Metabolite-Phosphopeptide Pairs

Prior to network construction, we first used the Mapman (Thimm et al., 2004) classification system to identify important functional categories in the interactions between metabolites and phosphopeptides. We scanned for positive interactions (i.e., concordant patterns over the four conditions), which we subsequently referred to as “connections” between metabolites and one or more phosphopeptide(s) of the respective investigated functional category (bin). Thus, the edges in this network were mainly based on the concordance index *I*_*C*_, which was developed for this purpose (Supplementary Table 8).

We only considered connections with sufficient evidence for a rank correlation and non-flat patterns (see methods; Supplementary Tables 9–11). As these filters already reduced the number of eligible interactions, we considered a concordance index *I*_*C*_≥6 as sufficient to robustly identify a connection. This choice of threshold value was based on balancing the necessity to reduce the chance of identifying co-expression pairs as valid connections whose patterns only matched by chance (i.e., false positives), with the prerequisite to include as many connections with truly matching patterns as possible (i.e., minimizing false negatives). Based on the comparison with the randomized data sets (Figure 4), a cut-off score of *I*_*C*_≥6 resulted in <2.5% of interactions having an *I*_*C*_ equal to or higher 6 in the simulated data for all the genotypes and simulation types. In the experimental data set, however, at least two times as many interactions had an *I*_*C*_≥6 in the wild type for all simulation types (Figure 4; Supplementary Table 9). Also, the proportion of patterns identified as “flat” by the Kruskal-Wallis test for any of the interaction partners was relatively low when using this threshold as a filter for the interaction table, with about 10% (wild type), 12% (*pgm* mutant), and 6% (*sweet11/12* mutant) of “flat” patterns among the co-expression pairs with *I*_*C*_≥6 (Supplementary Figure 3).

**Figure 7:**
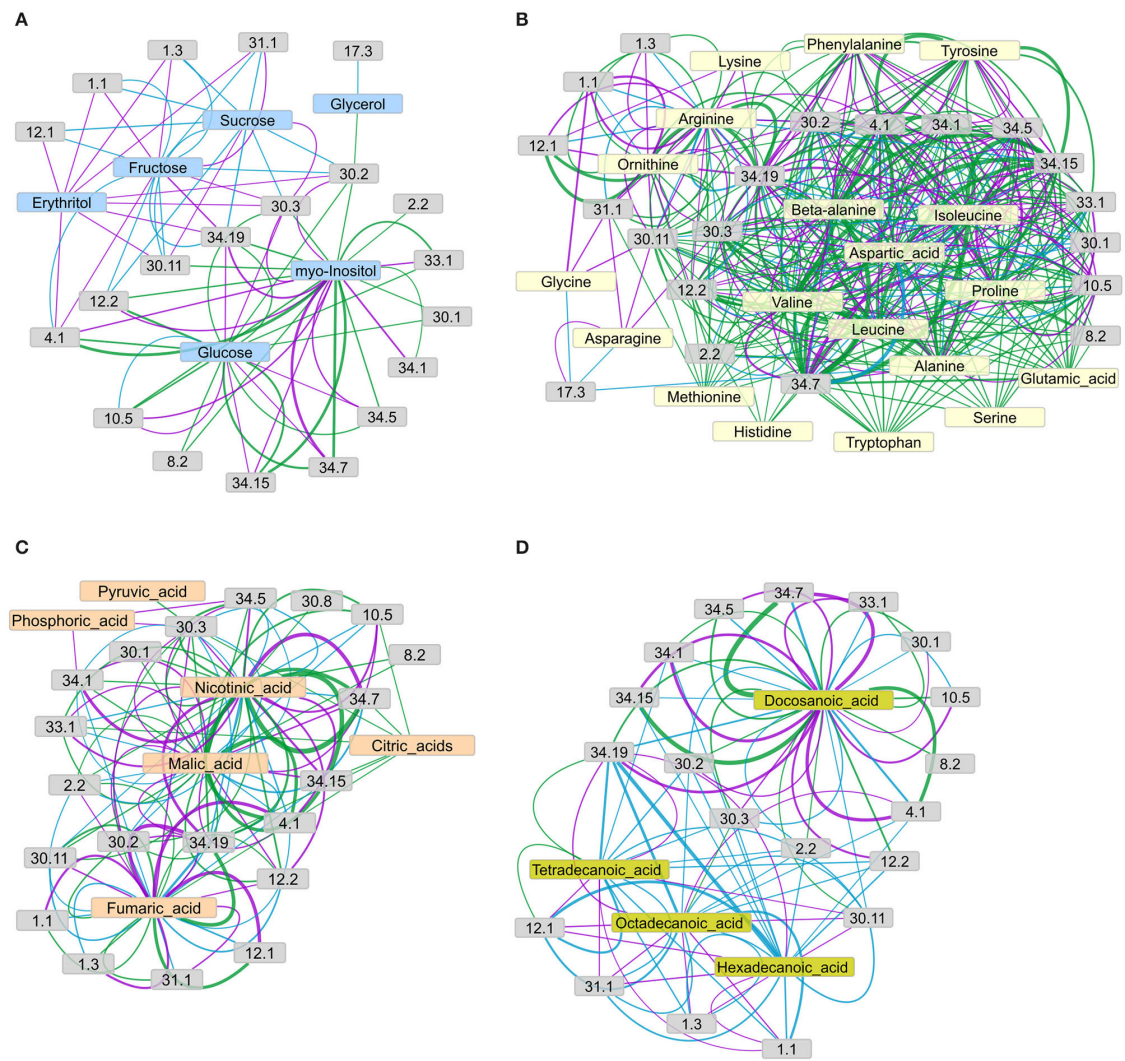
Networks of connections between metabolite concentration levels and protein phosphorylation, grouped by functional categories (bins), as suggested by the *I*_*C*_ for **(A)** sugars, **(B)** amino acids, **(C)** organic acids, and **(D)** fatty acids and sterols. The concordance index *I*_*C*_ of each metabolite-bin connection, represented by edge width, is calculated as the mean of the *I*_*C*_ values of all connections from the metabolites to the phosphopeptides in the respective functional group (according to Mapman), given that the connection's *I*_*C*_ value matches or exceeds the chosen threshold of *I*_*C*_ = 7. Edge color represents the connections revealed in each genotype: green = wild type, purple = *pgm* mutant, cyan = *sweet11/12* mutant. Node description according to Mapman: 1.1 photosynthesis.light reactions; 1.3 photosynthesis.Calvin-Benson cycle; 2.2 major carbohydrate metabolism.degradation; 4.1 glycolysis.cytosolic branch; 12.1 N-metabolism.nitrate; 29.2 protein.synthesis; 29.4 protein.posttranslational modification; 29.5 protein.degradation; 30.1 signaling.sugar and nutrient physiology; 30.2 signaling.receptor kinases; 30.3 signaling.calcium; 30.11 signaling.light; 31.1 cell.organization; 34.1 transport.p- and v-ATPases; 34.5 transport.ammonium; 34.7 transport.phosphate; 34.19 transport.aquaporins; 33.99 development.unspecific (contains SWEET proteins). Network layouts were obtained with an Allegro-Fruchterman Reingold algorithm using concordance indices *I*_*C*_ as edge-weighting parameter.

Higher *I*_*C*_ thresholds resulted in lower number of interactions but higher confidence in their biological relevance, thus increasing the likelihood of false negatives while reducing false positives. For example, applying a threshold of *I*_*C*_≥7 in the wild type decreased the proportion of positive interactions from 4.6 to 2.9%, but at the same time, the ratio of co-expression pairs in experimental vs. simulated data increased by about 7–13% while decreasing the share of “flat” patterns from 9.8 to 4.4% (Supplementary Table 9; Supplementary Figure 3). Using an even stricter threshold, *I*_*C*_≥10, resulted in only 0.66% of all interactions being classified as relevant; however, none of these had a flat co-expression partner (brown circles in Supplementary Figure 3), and the ratio of co-expression pairs in experimental vs. simulated data increased from 34 to 70% compared to *I*_*C*_≥6 in the wild type (Supplementary Table 4). In all the genotypes, the concordance index was particularly suited to reduce the proportion of “flat” patterns, while the Euclidean distance of z-scores squarely failed, as the standardization transformed “flat” patterns to patterns with a standard deviation of 1 (Supplementary Figure 3). Rank correlation coefficients were also able to filter out “flat” patterns to some extent but less efficiently than the *I*_*C*_. Thus, for the construction of metabolite–phosphopeptide networks, we applied the stricter threshold of *I*_*C*_≥7 (Figure 7). Significant Spearman's ρ of the connection and non-flat patterns for both partners were made additional requirements.

In the network of sugars and sugar alcohols (Figure 7A), glucose and *myo*-inositol were the most connected (highest degree) metabolites in the wild type, forming connections to glycolysis (bin 4.1), TCA cycle (bin 8.2), cell wall (bin 10.5), ammonia metabolism (bin 12.2), signaling (bins 30.1, 30.2, and 30.3), development (bin 33.1), and transport (bins 34.5, 34.7, 34.15, and 34.19). The other sugars and sugar alcohols in the wild type did not show any major connections to functional categories, with the exception of glycerol, which connected to receptor kinase signaling (bin 30.2). However, in the mutants, this picture drastically changed. In the *pgm* mutant, fructose and erythritol were the most connected metabolites, showing strong connections to aquaporins (bin 34.19) and vesicle trafficking (bin 31.1) and weaker connections to photosynthesis light reactions (bin 1.1), Calvin-Benson Cycle (bin 1.3), glycolysis (bin 4.1), and calcium signaling (bin 30.3). The sucrose in the *pgm* mutant was connected to calcium signaling (bin 30.3) and light signaling (bin 30.11). However, in the *pgm* mutant, the number of connections for glucose was highly reduced, and the remaining ones had lower *I*_*C*_. In the *sweet11/12* mutant, the network revealed only few significant edges connecting both sucrose and fructose with photosynthesis (bins 1.1 and 1.3), N-metabolism (bin 12.1 and 12.2) receptor kinase signaling (bin 30.2), light signaling (bin 30.11), vesicle trafficking (bin 31.1), and aquaporins (bin 34.19). These observations indicate a shift in centrality from glucose to fructose and sucrose when comparing the wild type to the *pgm* and *sweet11/12* mutants. Sucrose, whose concentration in the leaves was higher in the mutants, became more connected in the network, with strong connections to a number of functions such as N-metabolism ammonia (bin 12.2), receptor kinase signaling (bin 30.2), and aquaporins (bin 34.19).

In general, the amino acids (Figure 7B) showed a large number of connections to various functional categories, revealing substantial differences between the genotypes. The number of connections was largest in the wild type and decreased for both mutants. The average *I*_*C*_ value of all the connections decreased from the wild type to the *pgm* and *sweet11/12* mutants (Supplementary Table 8). Amino acids with functional connections in all the genotypes were arginine, aspartic acid, isoleucine, valine, and, to a lesser, extent alanine, beta-alanine, and ornithine, which only had few connections. Functional categories connected to at least two of the amino acids in all the genotypes were photosynthesis light reactions (bin 1.1), glycolysis (bin 4.1), cell wall proteins (bin 10.5), receptor kinase signaling (bin 30.2), calcium signaling (bin 30.3), and transport (bins 34.1, 34.5, 34.7, and 34.19). In the wild type, most of the amino acids formed a dense network connected to the processes mentioned above, and these connections showed strong support with a large *I*_*C*_ value. In the *pgm* mutant, however, the density of the network was already reduced. For glutamic acid, histidine, methionine, serine, and tryptophan, we did not find any connections to functional categories with a large-enough *I*_*C*_ value in the *pgm* mutant. On the other hand, new connections for asparagine, glycine, and lysine with light signaling (bin 30.11), photosynthesis light reactions (bin 1.1), and vesicle trafficking (bin 31.1) were found in this mutant, which was not apparent in the wild type. The density of the network was further reduced in the *sweet11/12* mutant, where only six amino acids with at least two connections each remained. The most prominent connections were those of aspartic acid, isoleucine, and valine with glycolysis (bin 4.1) and aquaporins (bin 34.19).

The network of organic acids (Figure 7C) around fumaric acid, malic acid, nicotinic acid, pyruvic acid, and citric acids showed large differences between the wild type and the mutants. In the wild type, fumaric acid, malic acid, and nicotinic acid showed the strongest connections with glycolysis (bin 4.1) and transport processes (phosphate transport, bin 34.7; potassium transport, bin 34.15; aquaporins, bin 34.19). In the *pgm* mutant, fumaric acid and nicotinic acid remained with strong connections, while the connection of malic acid with glycolysis or transport processes was less pronounced. In the *sweet11/12* mutant, organic acids were only weakly (low *I*_*C*_ values) connected to functional categories. Putrescine was found as a highly connected metabolite with the strongest connection to nitrate metabolism (bin 12.1) and aquaporins (bin 34.19) in all the genotypes.

The network of fatty acids (Figure 7D) showed the opposite picture compared to the amino acid network, as network density was low in the wild type and denser in the mutants, especially in the *sweet11/12* mutant. Docosanoic acid was the only fatty acid that, in all the genotypes, was strongly connected with cellular, such as glycolysis (bin 4.1), cell wall proteins (bin 10.5), signaling (bins 30.1, 30.2, and 30.3), and transport processes (proton ATPases, bins 34.1; ammonium transport, bin 34.5; phosphate transport, bin 34.7; potassium transport, bin 34.15; aquaporins, bin 14.19). Tetradecanoic acid was connected to nitrate metabolism (bin12.1) and aquaporins (bin 34.19) in all the genotypes. In the *pgm* mutant, connections of functional categories to docosanoic acid remained similar compared to the wild type, while tetradecanoic acid, hexadecanoic acid, and octadecanoic acid showed additional connections to aquaporins (bin 34.19), nitrate metabolism (bin 12.1), vesicle trafficking (bin 31.1), and photosynthesis (bins 1.1 and 1.3), which were not present in the wild type. Fatty acids in the *sweet11/12* mutant formed a very dense network in which carbohydrate metabolism (bin 2.2), receptor kinase signaling (bin 30.2), light signaling (bin 30.11), and aquaporins (bin 34.19) were connected to all four fatty acids. Phosphopeptides of photosynthetic proteins (bins 1.1 and 1.3), N-metabolism (nitrate assimilation, bin 12.1; ammonium assimilation; bin 12.2), and proteins of vesicle trafficking (bin 31.1) again formed a separate network from docosanoic acid, which was not found in the wild type.

### Selected Phosphopeptides With Strong Connection to Metabolites

In the wild type, we found that phosphopeptides from proteins in the functional category of phosphate transporters (bin 34.7), a doubly phosphorylated peptide, (SLEEL(pS)GEAEV(pS)HDEK) from PHT1;2, showed strongest connection to metabolites, in particular to organic acids and some amino acids (Figures 7B–D). The phosphopeptide SDKPLNY(pS)PDPENESGINER from the potassium transporter KUP8 showed strongest connections to amino acids valine and nicotinic acid. Overall, aquaporins, specifically PIP1;1, PIP1;2, PIP2;2, PIP2;4; PIP2;6, PIP2;7, PIP2;8 (bin 34.19), were one of the two functional groups with the most pronounced overrepresentation among metabolite–phosphopeptide interactions (the other group was bin 10.5, cell wall proteins, Supplementary Table 3, ST3E). This was likely due to aquaporins being phosphorylated at multiple phosphorylation sites for membrane targeting and regulation (Tornroth-Horsefield et al., 2006; Prak et al., 2008). Increased phosphorylation of aquaporins has been previously shown in Arabidopsis roots in response to external sucrose supply (Wu et al., 2013). Thus, it was not surprising to find a connection of sucrose with aquaporin phosphopeptides in the *sweet11/12* mutant, which showed the strongest variations in internal sucrose concentrations. In all the three genotypes, the phosphopeptides of aquaporins were strongly connected to the fatty acids and the organic acids fumaric acid, nicotinic acid, and malic acid (Supplementary Tables 9–11).

Among the signaling proteins, the phosphopeptide SE(pS)LGHR(pS)DV(pS)(pS)PEAK of KING1 (AT3G48530), the SNF1 regulatory subunit gamma 1, had the strongest connection to docosanoic acid in all the genotypes, with increasing *I*_*C*_ values in *pgm* and *sweet11/12*. At the same time, the *I*_*C*_value of the connection of KING1 with sucrose was rather low in the wild type (1.75) and was further decreased in the *pgm* mutant (−1.75) and the *sweet11/12* mutant (−6.5). These findings at the individual phosphopeptide level are supported by the increasingly dense network of fatty acids in the *sweet11/12* mutant (Figure 7D) and suggest a strong involvement of fatty acids in cellular signaling, especially under conditions when sucrose levels are deregulated because of metabolic manipulation. Among the phosphopeptides from proteins with functions in light signaling (bin 30.11), the phosphopeptide GT(pS)PQPRPQQEPAPSNPVR of photoreceptor PHOT1 was among the strongly connected individual phosphopeptides. It showed particularly strong connections in the *sweet11/12* mutant, in which strong positive connections of PHOT1 with the long-chain fatty acids, such as tetradecanoic acid, hexadecanoic acid, and octadecanoic acid, existed, and further connections with the sugars sucrose and fructose were found (Supplementary Table 11).

Two isoforms of nitrate reductase (AT1G77760 and AT1G37130) were found with phosphopeptides containing the well-known regulatory phosphorylation site that deactivates a nitrate reductase when phosphorylated (Sanchez and Heldt, 1990). These phosphopeptides showed strong connections (large *I*_*C*_values) with sucrose in the *sweet11/12* mutant but lower *I*_*C*_values in the *pgm* mutant and the wild type. Phosphorylation of nitrate reductases did not show a concordant behavior (strongly negative *I*_*C*_values) toward docosanoic acid in either genotype. Instead, the phosphopeptides of nitrate metabolism (bin 12.1), with SV(pS)SPFMNTASK and SV(pS)TPFMNTTAK of nitrate reductases NIA1 and NIA2, were connected to a number of organic acids (docosanoic acid, nicotinic acid, malic acid, and fumaric acid) and amino acids (valine, isoleucine, and aspartic acid) in all the genotypes.

Other functional groups that were substantially overrepresented among the phosphopeptides with strong connections to metabolites in all the genotypes were proteins from photosynthesis light reactions (bin 1.1) and Calvin-Benson Cycle (bin 1.3). The most highly connected phosphopeptides were (pT)AILERR from PSBA, a chlorophyll-binding protein D1, and EHGN(pS)PGYYDGR and KFETLSYLPDLTD(pS)ELAK from RBCS1A, the small subunit of RuBisCO (Supplementary Tables 9–11). These phosphopeptides showed strongest connections (i.e. highest *I*_*C*_ values) to sucrose in the *pgm* and *sweet11/12* mutants.

The most highly connected phosphopeptides from carbohydrate metabolism (bin 2.2) were AAAAS(pS)DVEEVKTEK from a fructokinase, seven peptides from the beta-amylase BAM1, and several different phosphopeptides from the cytosolic inveratase CINV1. We found only two metabolites (putrescine and fumaric acid) with a direct connection to these phosphopeptides that existed in all the genotypes. In the *pgm* mutant, no other metabolites were connected to carbohydrate metabolism. In the glycolysis pathway (bin 4.1), the phosphopeptides with strongest connections to metabolites were (pS)AQELVK of PPC3, ATGAFILTA(pS)HNPGGPTEDFGIK of cytosolic phosphoglucomutase (in all genotypes; see Supplementary Tables 9–11), and NSEDSGVTVDGS(pS)PSAK of fructose 2,6 bisphosphate phosphatase (especially in *sweet11/12*; see Supplementary Table 11). The long-chain fatty acids hexadecanoic acid and octadecanoic acid showed a connection to phosphopeptides from glycolysis only in the mutants.

## Discussion

Networks are a powerful tool to understand regulatory processes in biological systems (Rosato et al., 2018). A common starting point for network inference is the calculation of the correlation for each pair of nodes (i.e., in our case, each metabolite-phosphopeptide pair) as a measure of co-expression (Song et al., 2012; Saint-Antoine and Singh, 2020). Such networks will have the potential to yield powerful biological insights (Song et al., 2012) and define nodes with importance in information flow through the network by path analysis (Gilbert et al., 2021).

In this study, rather than attempting to extract causal directions or distinguishing between direct and indirect regulation processes, we developed an alternative co-expression measure, the “concordance index”, which quantifies analogous (“concordant”) patterns of a metabolite-phosphopeptide pair. We complemented this approach by applying further filters, using more traditional co-expression measures such as the Spearman correlation. Given the structure of our data sets with four to six measurements for each of just four conditions, we found that the concordance index was best suited to capture the relationship between metabolites and phosphopeptides. “Flat” concentration/intensity patterns were filtered out well, and pairs where a high degree of co-expression existed were most clearly distinguished by this index. Also, the concordance index was less prone to overestimating co-expression than the correlation measures in cases where the mean values of at least two conditions were similar. We did not use mutual information measures, as they are usually better suited to discrete or categorical variables and can safely be replaced by correlation measures in case of general monotonic relationships between continuous variables (Song et al., 2012). We used the value of the concordance index both as an indication of the connection strength of the corresponding metabolite-phosphopeptide pair and as a filter by choosing a cut-off value that defines whether a connection is further considered or not. We defined the applied threshold values based on the balance of false-positives and false-negatives as estimated from the simulated data set.

In addition to using the concordance index as a measure of co-expression for inferring networks, we used different clustering algorithms to group the metabolites and phosphopeptides into clusters. The results were more robust for the metabolites, as the phosphopeptides formed a relatively spherical point cloud in the (DR, DS, NR, NS)-space. Assigning metabolites to clusters that were qualitatively different between the genotypes, therefore, indicated that each of these metabolites showed a different abundance pattern across conditions when sucrose metabolism or sucrose export was disturbed. This approach proved to add complementary information to the co-expression networks, as it focused more on qualitative changes between patterns as opposed to hard thresholds for connections.

To reduce the level of complexity when constructing co-expression networks, we grouped the phosphopeptides according to the classification by Mapman (Thimm et al., 2004) rather than directly constructing a full network of all the metabolites and phosphopeptides. This also helped us to identify functional categories of particular prominence in the data set. An earlier study on dissecting the subcellular compartmentation of proteins and metabolites in Arabidopsis leaves by non-aqueous fractionation revealed a tight physical connection of metabolic enzymes with metabolites of the respective pathway (Arrivault et al., 2014). This could be taken as an indication that many of the connections identified for enzymes of the primary metabolism indeed are based on direct interactions in a pathway context rather than indirect relationships.

### Regulatory Modules of Phosphopeptides and Metabolites

Our analysis describes connections of metabolites with phosphopeptides of metabolic enzymes, transporters, or regulatory proteins (e.g., kinases). Therefore, we used the thorough methods discussed above to robustly derive these connections, but the data set was not suitable to derive causality. Thus, it remains subject to further research as to whether metabolites caused changes in respective phosphopeptide levels or whether proteins regulated by phosphorylation affected the synthesis/degradation of the metabolites. We likely identified examples for both possibilities. The sucrose non-fermenting-related kinase 1 (SnRK1) is an enzyme complex that has been widely discussed to have roles in sucrose sensing (Rolland et al., 2002) and is well-established as a central regulator of cellular metabolism (Wurzinger et al., 2018). Catalytic subunits are activated by phosphorylation in the activation loop (Shen et al., 2009; Glab et al., 2017). Isoforms of SnRK1 were shown to play a role in stress signaling (abscisic acid signaling) and metabolic signaling, especially sugars (Radchuk et al., 2009; Cho et al., 2012; Tsai and Gazzarrini, 2012). In this study, we found the regulatory subunit KING1 to have strong connections to different metabolites, sucrose among them, for which connection strength (measured by the concordance index) was altered in the different genotypes. This could be an excellent example, where indeed metabolite levels (e.g., sucrose or fatty acids) could affect the phosphorylation status of the regulatory subunit and, thus, affect kinase activity. Indeed, the phosphorylation status of KIN10, an isoform of the catalytic subunit of SnRK1, was found to have lower abundance at the end of the day in the shoots of the *pgm* and *sweet11/12* mutants (Figure 6C). In turn, SnRK1 was proposed as the primary kinase to phosphorylate nitrate reductase (Harthill et al., 2006). Again, strong differences in the connection of metabolites (e.g., sucrose) with nitrate reductase were observed among the genotypes. In this case, the activity of nitrate reductase likely affected metabolite pools (e.g., amino acids and organic acids) at different levels in the genotypes analyzed and resulted in different metabolite patterns in the respective genotypes. Thus, the enzyme complex of SnRK1 has been confirmed as a key regulator of metabolism in our study.

### Fatty Acids as Putative Signaling Molecules and/or Overflow Metabolites

It is noteworthy that fatty acids, especially long-chain fatty acids (such as tetradecanoic acid, hexadecanoic acid, octadecanoic acid, and docosanoic acid), were found as highly connected metabolites, especially in genotypes with disturbed sucrose metabolism (*pgm* mutant) or transport (*sweet11/12* mutant). Very long-chain fatty acids, formally defined as fatty acids with more than 18 carbons, were indeed previously identified as responsive to various stress signals (De Bigault Du Granrut and Cacas, 2016) and, thus, were proposed as cellular signaling molecules. They are suggested to act either by direct release from membranous context, through vesicle trafficking or through membrane microdomains (De Bigault Du Granrut and Cacas, 2016). However, the effect of altered fatty acid levels may also be indirect. Fatty acids are important constituents of photosynthetic lamellae; therefore, lipid composition will affect photochemistry. Thus, a change in lipid composition is expected to result in alterations in photosynthesis and related metabolism (as has been observed, e.g., in the mutants). Regulation of membrane fatty acid composition is actively adjusted, for example, in response to temperature changes (Falcone et al., 2004), but this refers mainly to changes in desaturation status not due to alterations in the length of fatty acids. Interestingly, a link between lipids and sucrose exists during germination of the plants, when lipids from oil bodies are degraded to feed sucrose synthesis. Deficiencies in lipid degradation affect sucrose levels (Cui et al., 2016). Thus, likely altered long-chain fatty acid levels in the *sweet11/12* mutant could be due to increased fatty acid synthesis in response to elevated sucrose levels in the leaves to dispose excess carbon.

## Conclusions

We developed a robust approach to identify concordance between metabolites and phosphopeptides in order to construct a complex regulatory network between metabolites and functional categories (bins). This network was highly dynamic in genotypes with altered cellular sucrose levels. In this network, we identified KING1, the regulatory subunit of SnRK1, as a major regulator connecting metabolism with enzyme activities through the targets of SnRK1. The network also revealed strong changes in fatty acid metabolism, especially in the *sweet11/12* mutant. This may represent a combination of fatty acid signaling and metabolic overflow reactions due to high sucrose concentrations. Taken together, our approach, especially by including three different genotypes, provides novel protein-metabolite relationships to be explored in future targeted research.

## Data Availability Statement

The datasets presented in this study can be found in online repositories. The names of the repository/repositories and accession number(s) can be found below: https://www.ebi.ac.uk/pride/archive/, PXD031942.

## Author Contributions

XW, YZ, and AF generated the data. TS analyzed the data. WS and TS wrote the paper and interpreted the results. All authors contributed to the article and approved the submitted version.

## Conflict of Interest

The authors declare that the research was conducted in the absence of any commercial or financial relationships that could be construed as a potential conflict of interest.

## Publisher's Note

All claims expressed in this article are solely those of the authors and do not necessarily represent those of their affiliated organizations, or those of the publisher, the editors and the reviewers. Any product that may be evaluated in this article, or claim that may be made by its manufacturer, is not guaranteed or endorsed by the publisher.
