# Supplementary figures and images for: Regulatory Modules of Metabolites and Protein Phosphorylation in Arabidopsis Genotypes With Altered Sucrose Allocation
Source: Front Plant Sci. 2022 May 19;13:891405. doi: 10.3389/fpls.2022.891405 (PMC9161306; doi:10.3389/fpls.2022.891405)

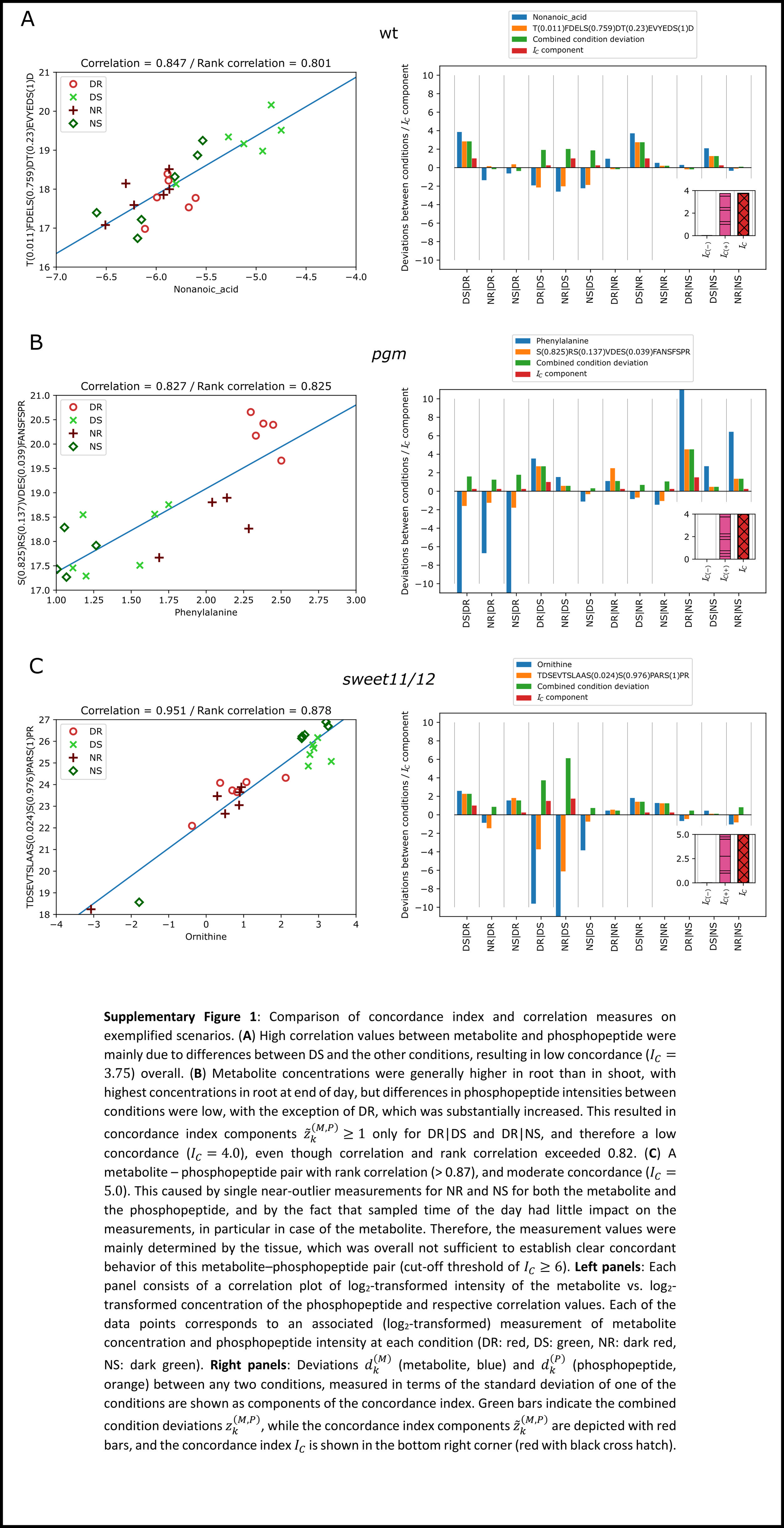

Supplement: Supplementary file 12 [file Image_1.TIF]

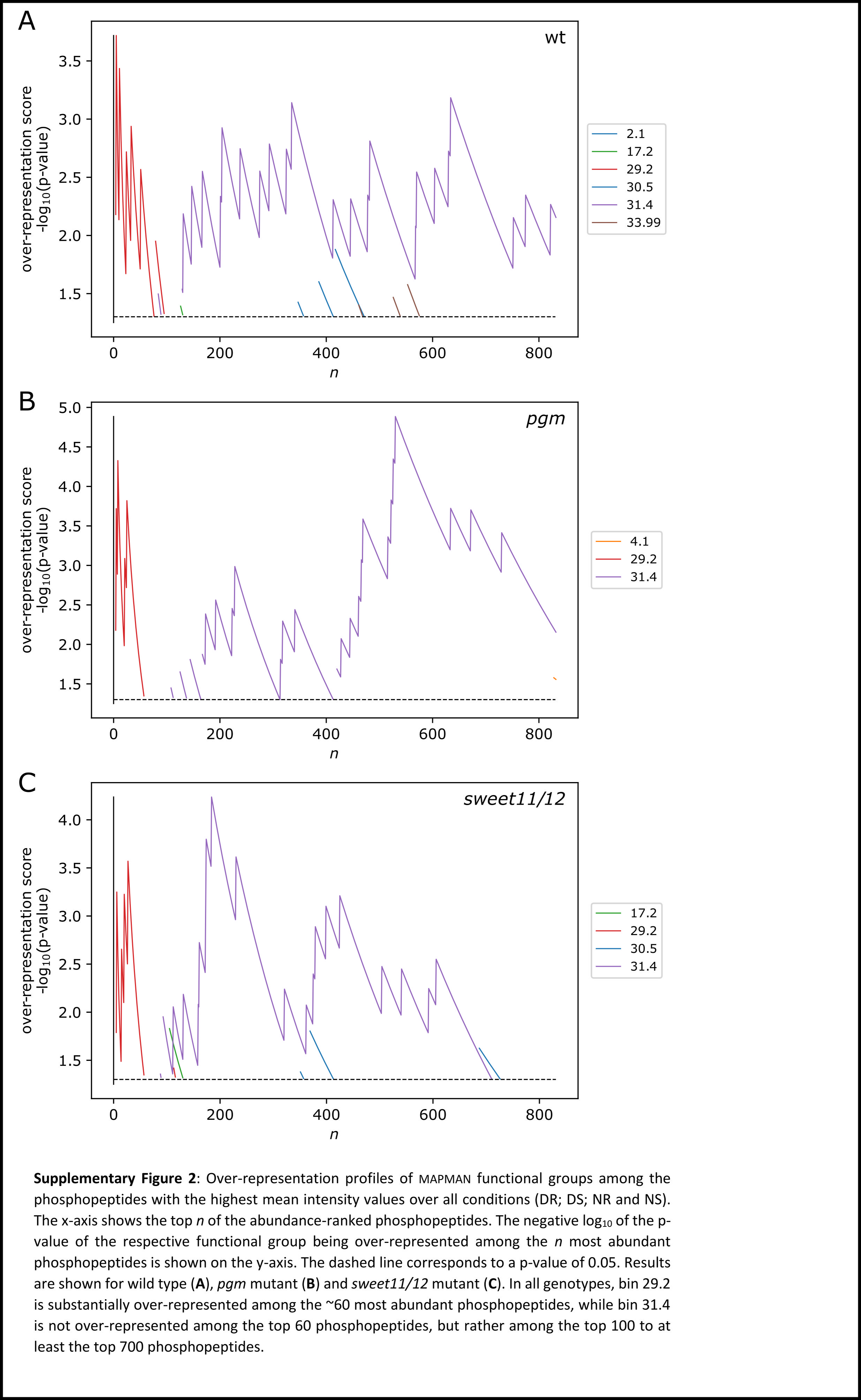

Supplement: Supplementary file 13 [file Image_2.TIF]

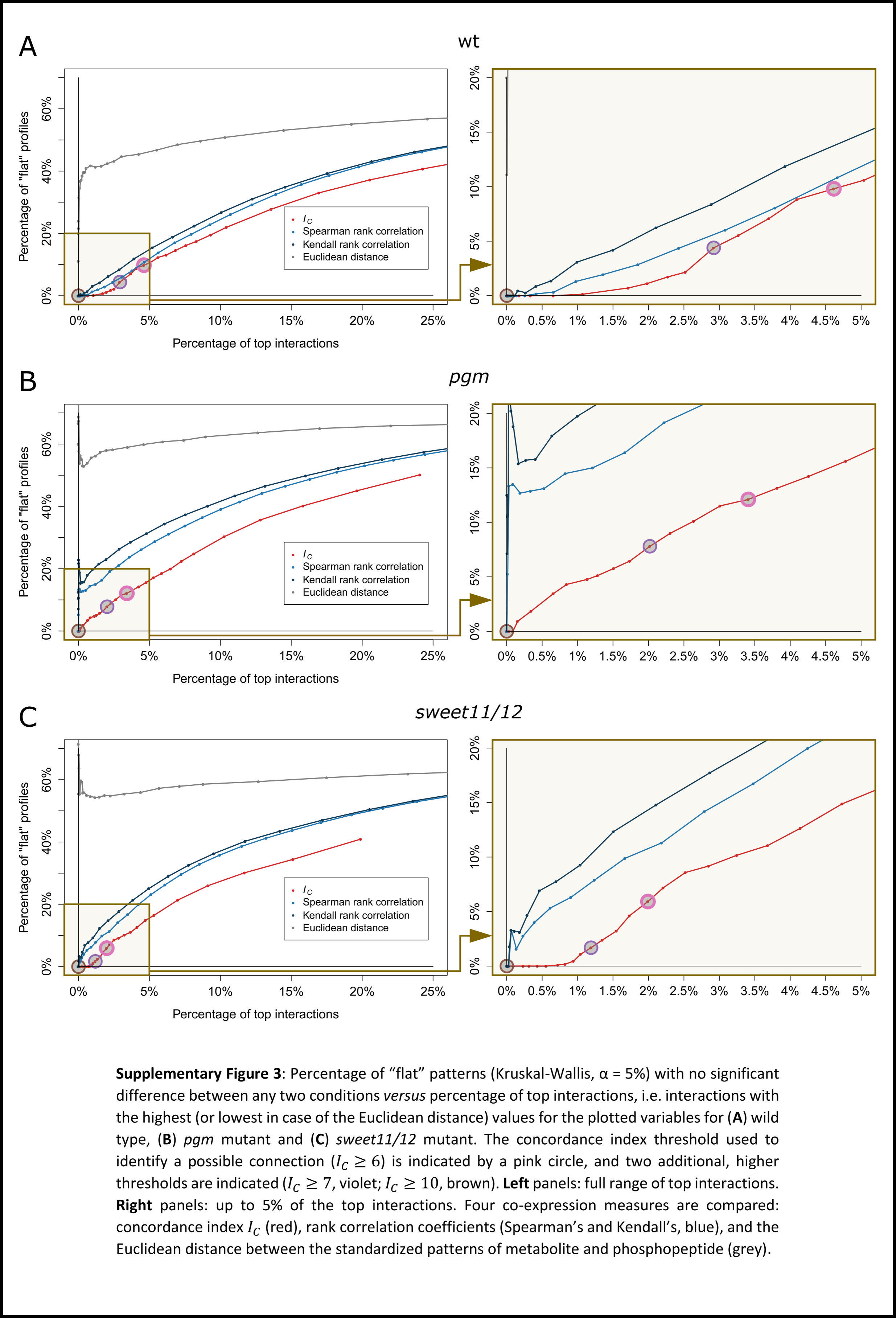

Supplement: Supplementary file 14 [file Image_3.TIF]
